# Targeting of Telomeric Repeat-Containing RNA G-Quadruplexes: From Screening to Biophysical and Biological Characterization of a New Hit Compound

**DOI:** 10.3390/ijms221910315

**Published:** 2021-09-24

**Authors:** Simona Marzano, Bruno Pagano, Nunzia Iaccarino, Anna Di Porzio, Stefano De Tito, Eleonora Vertecchi, Erica Salvati, Antonio Randazzo, Jussara Amato

**Affiliations:** 1 Department of Pharmacy, University of Naples Federico II, Via D. Montesano 49, 80131 Naples, Italy; simona.marzano@unina.it (S.M.); bruno.pagano@unina.it (B.P.); nunzia.iaccarino@unina.it (N.I.); anna.diporzio@unina.it (A.D.P.); antonio.randazzo@unina.it (A.R.); 2 Molecular Cell Biology of Autophagy, The Francis Crick Institute, 1 Midland Road, London NW1 1AT, UK; stefano.de-tito@crick.ac.uk; 3 Institute of Experimental Endocrinology and Oncology, National Research Council, 80131 Naples, Italy; 4 c/o Department of Biology and Biotechnology “C. Darwin”, Institute of Molecular Biology and Pathology, National Research Council, Sapienza University of Rome, Via degli Apuli 4, 00185 Rome, Italy; eleonora.vertecchi@uniroma1.it (E.V.); erica.salvati@cnr.it (E.S.)

**Keywords:** TERRA G-quadruplex, drug discovery, biophysical characterization, conformation-selective ligand, in vitro biological assays

## Abstract

DNA G-quadruplex (G4) structures, either within gene promoter sequences or at telomeres, have been extensively investigated as potential small-molecule therapeutic targets. However, although G4s forming at the telomeric DNA have been extensively investigated as anticancer targets, few studies focus on the telomeric repeat-containing RNA (TERRA), transcribed from telomeres, as potential pharmacological targets. Here, a virtual screening approach to identify a library of drug-like putative TERRA G4 binders, in tandem with circular dichroism melting assay to study their TERRA G4-stabilizing properties, led to the identification of a new hit compound. The affinity of this compound for TERRA RNA and some DNA G4s was analyzed through several biophysical techniques and its biological activity investigated in terms of antiproliferative effect, DNA damage response (DDR) activation, and TERRA RNA expression in high vs. low TERRA-expressing human cancer cells. The selected hit showed good affinity for TERRA G4 and no binding to double-stranded DNA. In addition, biological assays showed that this compound is endowed with a preferential cytotoxic effect on high TERRA-expressing cells, where it induces a DDR at telomeres, probably by displacing TERRA from telomeres. Our studies demonstrate that the identification of TERRA G4-targeting drugs with potential pharmacological effects is achievable, shedding light on new perspectives aimed at discovering new anticancer agents targeting these G4 structures.

## 1. Introduction

G-quadruplexes (G4s) are higher-order noncanonical nucleic acid structures formed by guanine-rich sequences [1]. These structures exhibit a common stem arrangement of stacked G-tetrads, where four guanine bases associate through Hoogsteen-type hydrogen bonding and their oxygen O6 atoms are arranged to coordinate metal cations, such as potassium or sodium, to give stability to the whole structure [1]. G4s can be intramolecular, i.e., formed by a single nucleic acid molecule, or intermolecular, i.e., formed by two or four strands [1]. Generally, potential intramolecular G4s have a consensus sequence of G_≥3_N_1–7_G_≥3_N_1–7_G_≥3_N_1–7_G_≥3_, where N is any nucleotide, even if some non-consensus sequences were reported to fold into stable G4s [2,3]. Sequences between G-tracts are variable and, depending on their length and composition, determine different loop conformation and the overall G4 topology, which can be parallel, antiparallel, or hybrid [4,5].

Accumulating evidence shows that DNA and RNA G4 structures are formed in vivo [6,7,8,9,10,11,12,13], and play pivotal roles in regulating DNA transcription and replication, RNA translation, and the maintenance of genome integrity [14,15,16,17,18]. Indeed, G4-forming sequences are particularly enriched in proto-oncogene promoters [19], origins of replication [20], 5′- and 3′-untranslated regions (UTRs) of mRNA of a large number of genes [21], and at the ends of human chromosomes, the telomeres [22,23].

Telomeres are specialized nucleoprotein structures that protect chromosomal DNA from progressive degradation and ensure the integrity of linear chromosomes by preventing the natural ends from being recognized as DNA damage [22,23]. Human telomeres terminate with a 3′ single-stranded DNA overhang, which is composed of tandem repeats of the short guanine-rich sequence d(TTAGGG) synthesized by telomerase, a telomere-specific reverse transcriptase. In normal human cells, telomeres progressively shorten, leading to growth arrest upon telomere uncapping known as replicative ageing. Conversely, telomerase is overactive in numerous cancer subtypes, contributing to the ability of these cells to indefinitely proliferate due to the lack of chromosomal shortening [24]. Mounting evidence shows that G4s formation and stabilization at telomeres inhibits telomerase activity and induces DNA damage response, leading to chromosomal shortening and cell death [25,26]. Therefore, designing small molecules able to bind and stabilize telomeric G4s represents a potential avenue for developing novel selective anticancer agents [27,28,29].

At telomeres, the 3′ single-stranded overhang is preceded by a double-stranded DNA region that is composed of the repeated sequences d(TTAGGG) on one strand and the complementary d(CCCTAA) repeats on the other [30]. The transcription of the telomeric C-rich strand in chromosomes produces telomeric repeat-containing RNA (TERRA), which has a canonical G-rich motif of sequence r(UUAGGG) [31,32,33]. As such, TERRA can fold into G4 structures. Besides regulating telomerase activity and protecting chromosome ends from telomere degradation, TERRA also takes part in heterochromatin formation and homologous recombination [34,35,36]. TERRA G4 structures are potentially more valuable therapeutic targets than their DNA counterparts, since telomere heterochromatin formation is required in all cancer cells, even in those that do not require telomerase to elongate their telomeres (ALT-positive tumors) [37]. These tumors display higher TERRA accumulation levels that appear to play a direct role in telomere elongation [38]. For this reason, the design of small molecules targeting TERRA G4s is attracting ever-increasing attention.

Structural analysis showed that TERRA RNA and its corresponding DNA sequences, which differ only for the presence of thymine instead of uracil bases and for deoxyribose sugars instead of ribose, can adopt different G4 topologies. For example, the 22-nucleotide-long telomeric DNA of sequence d[AGGG(TTAGGG)_3_] adopts an antiparallel-stranded G4 conformation in Na^+^-containing solution and a hybrid [3+1] (parallel/antiparallel-stranded) conformation in K^+^-containing solution [1,39], while corresponding RNA sequence r[AGGG(UUAGGG)_3_] folds into a parallel-stranded G4 structure in both Na^+^ and K^+^ solutions [40]. Direct evidence for the presence of parallel-stranded TERRA RNA G4s in living cells was also provided by Xu and coworkers by using a light-switching pyrene probe [41]. Further confirmation of their existence in vivo came from both optical imaging investigations performed with other small molecules [7,42,43] as well as with the BG4 antibody [6], and from sequencing-based methods [44,45].

Several studies suggested that RNA G4s are more compact and thermally stable than their DNA counterparts [46,47,48] due to the 2′-hydroxyl (2′-OH) group in the ribose sugar and the networks of water-mediated contacts within the grooves of RNA [49]. The presence of the 2′-OH group in the ribose also induces guanine bases to adopt the *anti* conformation in the G4 structure, which can thereby only be parallel [50,51]. In addition, the presence of the 2′-OH groups may interfere with the interaction of ligands with the loops of RNA G4 by reducing their depth and width [52], and/or affecting the π–π stacking surface of the external G-tetrads. Therefore, the presence of 2′-OH groups in RNA G4s could represent an important structural feature to be taken into account in the design of selective RNA G4-targeting ligands [6].

Di Antonio and coworkers showed that selective RNA vs. DNA G4 targeting can be achieved even by introducing small modifications into a generic G4 binder [53]. Indeed, pyridostatin is not able to discriminate between RNA and DNA G4s, while the carboxypyridostatin derivative has high preference for G4 RNA [53].

**Figure 1:**
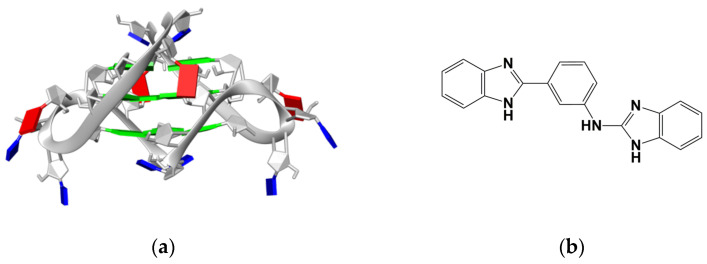
(**a**) Bimolecular G4 structure formed by TERRA RNA (guanine, green; adenine, red; uracil, blue). (**b**) Chemical structure of compound **BPBA**.

Herein, we identify new molecular scaffolds able to target *TERRA G4* by employing a strategy of high-throughput in silico screening of a large number of compounds from a commercially available database, followed by validation of the putative hits through a combination of experimental techniques. The selected 103 virtual screening-derived hits were experimentally evaluated for their binding properties by biophysical methodologies, identifying molecule N-[3-1H-1,3-benzodiazol-2-yl)phenyl]-1H-1,3-benzodiazol-2-amine (**BPBA**, Figure 1) as a promising hit compound able to bind and stabilize the G4 structure adopted by TERRA. The biophysical characterization of the binding profile of **BPBA** revealed that this ligand binds to parallel RNA and DNA G4 structures with a slightly higher affinity for the former, whereas it showed no affinity for double-stranded DNA.

In addition, the investigation of the biological activity of **BPBA** showed that it is particularly effective in high TERRA-expressing human cancer cells by binding and displacing TERRA from telomeres.

## 2. Results and Discussion

### 2.1. Compound Selection

Virtual-screening calculations were performed to identify drug-like molecules capable of binding to TERRA, using as target the three-dimensional G4 structures formed by the 12-nt r(UAGGGUUAGGGU) sequence (*TERRA G4*, Figure 1) [50,54]. We employed a receptor-based virtual-screening approach based on the identification of druggable RNA hot spots and molecular docking (see Section 3 for details). To improve the efficacy of the virtual screening, some precalculations to define a potential binding site on the RNA were performed. Since most true binding sites are cavities or regions that provide a large surface for favorable interactions, we looked for an accessible area on the target RNA structures that could form strong polar and nonpolar interactions with a putative ligand. These areas were detected by performing docking calculations with the AutoDock Vina [55] tool embedded in Mcule (https://mcule.com, accessed on 15 July 2017) using a set of small solvent molecules having different polarity [56]. Solvent molecules are used because, thanks to their small size, they can efficiently fit also in buried cavities. Potential hot spots were located where the top binding pose of at least three different molecules were overlapping.

Once RNA hot spot regions that could be targeted by putative ligands were identified, docking calculations, restricted to the identified binding sites, were carried out with AutoDock Vina by using a diverse set of 58870 commercially available compounds as a screening library. This virtual-screening process resulted in 103 drug-like compounds that were selected for further experimental investigations and purchased.

### 2.2. Circular Dichroism Screening

**Figure 2:**
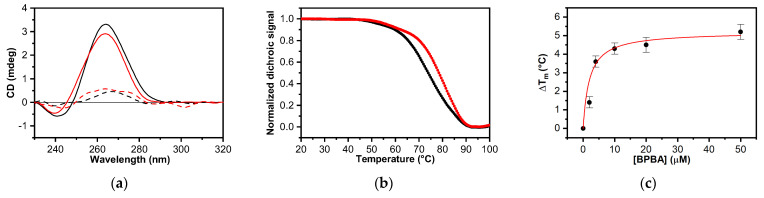
(**a**) CD spectra of *TERRA G4* in the absence (black line) and presence (red line) of 10 molar equiv of **BPBA** recorded at 20 and 100 °C (solid and dashed lines, respectively); (**b**) CD melting profiles of *TERRA G4* in the absence and presence (black and red dots, respectively) of 10 molar equiv of **BPBA** recorded at 1 °C/min heating rate; (**c**) CD stabilization curve for *TERRA G4* with **BPBA**.

In order to identify true hits, the 103 computationally selected small molecules were screened for their ability to thermally stabilize *TERRA G4* by using circular dichroism (CD) melting assay. Although CD is not usually used for large-scale screening purposes, it is widely employed to select nucleic acid-interacting compounds with high reliability [57,58]. Indeed, CD experiments require unmodified oligonucleotides, so changes in CD melting curves should only be produced by the direct interaction of the putative ligand with the nucleic acid structure. First, CD spectra were recorded to examine the potential of the selected compounds to eventually alter the dimeric propeller-type parallel conformation of *TERRA G4* in K^+^ buffer, whose CD spectrum in the absence of any compound resembles that reported in the literature, exhibiting a positive band at around 265 nm and a negative one at around 245 nm (Figure 2) [59]. RNA/ligand mixtures were obtained by adding putative ligands (10 molar equiv) to the folded *TERRA G4* structure. No significant variations of the CD profile were detected upon the addition of any compound (Figure S1), clearly suggesting they did not modify the parallel conformation adopted by *TERRA G4*. Then, the stabilizing properties of the compounds were evaluated by CD melting experiments measuring the ligand-induced change in the apparent melting temperature (Δ*T*_m_) of the G4 structure. Melting experiments in the absence and presence of each compound (10 molar equiv) were recorded by following the CD signal at the wavelength of the maximal intensity (265 nm) of *TERRA G4* (Figure S2). These experiments showed that 1 out of 103 tested compounds, namely, **BPBA** significantly increased the *T*_m_ of *TERRA G4* (Δ*T*_m_ = 4.5 (±0.4) °C, Figure 2 and Table 1). The remaining 102 compounds were unable to significantly stabilize such structure (Δ*T*_m_ < 3 °C) (Table S1), and thus were not further considered for the biophysical characterization of the interaction with *TERRA G4*.

Additionally, the investigation of *TERRA G4*/**BPBA** interaction was extended by performing CD melting experiments by using a range of ligand concentrations (Figure S3). The thermal-shift curves of the ligand followed a dose–response pattern (Figure 2c and Table S2), suggesting that the interaction of **BPBA** with G4 is specific.

### 2.3. BPBA Is a Selective G4 Binder That Exhibits Preference for Parallel G4 Conformations

Once a G4 ligand is validated, its selectivity towards a certain nucleic acid structure must be assessed. Under physiological conditions, most DNA is in the B form. Since binding to B-DNA duplex can cause undesired toxicity effects, it is crucial to determine the selectivity of the ligand towards G4 vs. duplex structures before proceeding with a more in-depth characterization of its binding properties. Here, a 20-mer hairpin-forming sequence (*Hrp*_20_) was chosen as a suitable duplex model. The CD spectrum of the hairpin in the presence of K^+^ was characterized by a positive band at around 280 nm and a negative one at 250 nm, confirming duplex formation (Figure S4). These bands were not modified upon the addition of compound **BPBA** (10 molar equiv; Figure S4). Next, CD melting experiments of the hairpin were recorded both in the absence and presence of ligand following variations in CD signal intensity at 280 nm. No significant change of *T*_m_ was observed in this case (Figure S4 and Table 1), suggesting that **BPBA** selectively stabilizes the G4 over duplex DNA conformation. 

**Figure 3:**
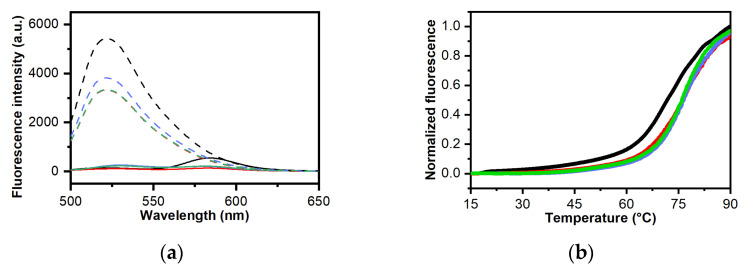
(**a**) Fluorescence emission spectra recorded at 15 °C (solid lines) and 90 °C (dashed lines); (**b**) FRET melting experiments for *F-TERRA-T* (0.2 μM) in the absence (black) and presence (red) of **BPBA** (2.0 μM). Experiments in the presence of **BPBA** were also performed by adding a large excess of *Hrp*_27_ duplex competitor (6.0 μM, blue; and 20.0 µM, green).

The RNA G4 stabilizing properties of **BPBA** were further investigated by Förster resonance energy transfer (FRET) melting assay using a G4-forming telomeric RNA sequence dually labeled with donor FAM and acceptor TAMRA at the 5′ and 3′ ends, respectively (*F-TERRA-T*) [60]. FRET melting curves (Figure 3) confirmed that **BPBA** is able to stabilize the G4 structure formed by telomeric RNA (Δ*T*_m_ = 3.4 (±0.1) °C). Moreover, to further confirm the selectivity of **BPBA** for G4 over the duplex, a competition FRET melting experiment was carried out in the presence of a large excess of a duplex model, i.e., a 27-mer hairpin duplex-forming DNA (*Hrp*_27_) [61,62,63,64]. The results of this experiment clearly showed that the stabilizing effect of **BPBA** on *TERRA G4* was not affected by the presence of the duplex competitor (Table 1), meaning that this compound preferably binds to G4s.

Since **BPBA** was selected for its ability to interact with *TERRA G4*, which adopts a parallel propeller-type conformation, we examined if **BPBA** also interacts with analogous parallel G4 structures formed by DNA and RNA G-rich sequences, and if it is potentially capable of discriminating between parallel and antiparallel G4 conformations. To this aim, we used three different G4-forming DNA sequences derived from the nuclease hypersensitive region of the *c-KIT* (*c-kit2 G4*) and *c-MYC* (*c-myc G4*) gene promoters, which form parallel propeller-type G4 structures in K^+^-containing buffer, and from the human telomeric DNA sequence, particularly the 23-mer truncation (*Tel*_23_ *G4*), which rather folds in an antiparallel (3 + 1) hybrid G4 conformation in the same buffer conditions [65,66,67,68]. To obtain information about the ability of **BPBA** to interact with other parallel RNA G4 structures, two additional G4-forming RNA sequences derived from the GSEC lncRNA (*GSEC G4*) and the 5′-UTR of Bcl-2 mRNA (*Bcl-2 G4*) were also investigated [69,70,71]. The proper folding adopted by each of these G4-forming sequences was first confirmed by CD spectra. As expected, *c-kit2 G4*, *c-myc G4, GSEC G4*, and *Bcl-2 G4* displayed a positive band at around 265 nm and a negative one around 240 nm in the CD spectrum (Figures S5 and S6). These bands are characteristic of parallel-stranded G4 topologies [59]. On the other hand, *Tel*_23_ *G4* showed a positive band at 289 nm with a shoulder at ca. 268 nm and a weak negative band at around 240 nm (Figures S5 and S6), which are consistent with the presence of a (3+1) hybrid G4 as major conformation [59]. As already done for the other nucleic acid molecules, CD experiments were also performed to examine the potential of **BPBA** to alter the native folding topology of these G4s. No significant variations in CD signal were observed for any of these G4 structures (Figures S5 and S6), suggesting an overall preservation of their G4 architectures upon addition of the ligand (10 molar equiv). Hence, the stabilizing properties of **BPBA** on these G4s were evaluated by CD melting experiments (Figures S5 and S6). Results of these experiments clearly indicate the ligand ability to bind and stabilize *c-kit2 G4* and *c-myc G4* (Δ*T*_m_ = 18.7 (±1.0) and 9.4 (±0.5) °C, respectively), both having parallel G4 conformations with a negligible effect (Δ*T*_m_ = 0.7 (±0.2) °C) on the *Tel*_23_ *G4* hybrid structure (Table 1). No relevant ligand-induced thermal shift was observed in the case of both *GSEC* and *Bcl-2 G4s* (Δ*T*_m_ < 3 °C), suggesting that **BPBA** could preferentially stabilize *TERRA G4* over other RNA G4s.

However, since the stabilization imparted by the ligand would naturally be more pronounced in intrinsically less stable oligonucleotides [72], the direct comparison of Δ*T*_m_ values cannot provide straightforward information on the binding affinity.

### 2.4. Analysis of Ligand Binding Affinity by Fluorescent Intercalator Displacement Assay

**Figure 4:**
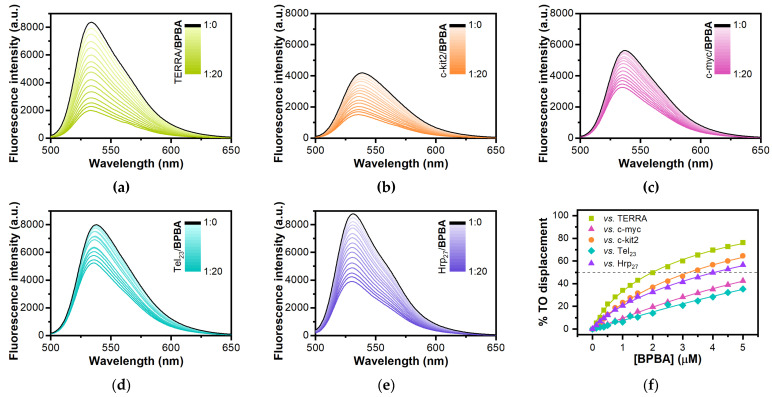
TO displacement titrations for (**a**) *TERRA G4*, (**b**) *c-kit2 G4*, (**c**) *c-myc G4,* (**d**) *Tel*_23_ *G4,* and (**e**) *Hrp*_27_ upon addition of increasing amounts of **BPBA**; (**f**) Dose–response curves from FID experiments.

To gain insight into the binding affinity of **BPBA** for the different RNA/DNA G4s, fluorescent intercalator displacement (FID) experiments were carried out. This assay is based on the competitive displacement of a light-up fluorescent probe, in this case, thiazole orange (TO), from the DNA upon addition of increasing amounts of a candidate ligand [73,74,75]. TO is almost nonfluorescent when free in solution, while it is strongly fluorescent when bound to DNA [75]. Ligand-induced TO displacement decreases fluorescence, thus allowing for the determination of their relative binding affinity for the structure under examination. Here, TO displacement by **BPBA** was investigated for *TERRA G4*, *c-kit2 G4*, *c-myc G4*, *Tel*_23_ *G4*, and *Hrp*_27_. **BPBA** concentrations required to give 50% TO displacement (DC_50_ values) were calculated from dose–response curves fitted to these data (Figure 4). As far as *TERRA G4* is concerned, the **BPBA** concentration at which 50% displacement was achieved was 2.4 (±0.4) μM, indicating good affinity for this G4 motif. On the other hand, a DC_50_ value of 3.8 (±0.6) and 4.1 (±0.5) μM was obtained for the interaction of **BPBA** with *c-kit2 G4* and *Hrp*_27_, respectively, suggesting a lower affinity than that for *TERRA G4*. In the case of *c-myc G4* and *Tel*_23_ *G4*, it was not possible to reach a 50% displacement of TO even after addition of a large excess of the binder (20 molar equiv), clearly suggesting weaker ligand interactions.

### 2.5. Study of Interaction between BPBA and TERRA G4 by Ethidium Bromide Displacement Assay

**Figure 5:**
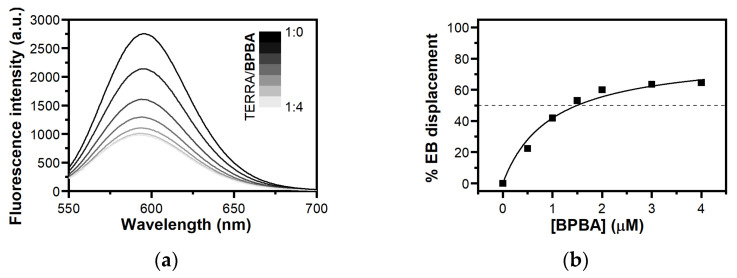
(**a**) Fluorescence spectra for ethidium bromide displacement from *TERRA G4* in the presence of increasing concentrations of **BPBA**; (**b**) Dose–response curves from FID experiments.

To obtain information on the binding mode of **BPBA** to *TERRA G4*, an ethidium bromide (EB) displacement assay was performed using fluorescence spectroscopy. EB binds to duplex DNA through intercalation, and to G4 DNA through π–π stacking on the external G-tetrads [76]. In the absence of DNA, EB emits weak fluorescence at 595 nm, while its fluorescence is strongly enhanced upon association with G4s as a consequence of the hydrophobic environment experienced by EB upon binding to the nucleic acid [77]. Therefore, the addition of a G4 ligand decreases EB fluorescence intensity if it binds to G4 via end-stacking mode. Displacement titrations performed by adding increasing amounts of **BPBA** to the *TERRA G4*/EB complex showed a substantial decrease in the fluorescence intensity of EB (Figure 5), thus suggesting an end-stacking binding mode for this ligand to *TERRA G4* [78,79]. In addition, the concentration of **BPBA** required to give the 50% decrease in EB fluorescence (DC_50_ value) was calculated from dose–response curves obtained by plotting the percentage of EB displacement against ligand concentration. A DC_50_ value of 1.3 (±0.4) µM was determined, confirming once again the strong interaction between **BPBA** and this G4 motif.

### 2.6. Determination of BPBA Affinity for RNA and DNA G4s

**Figure 6:**
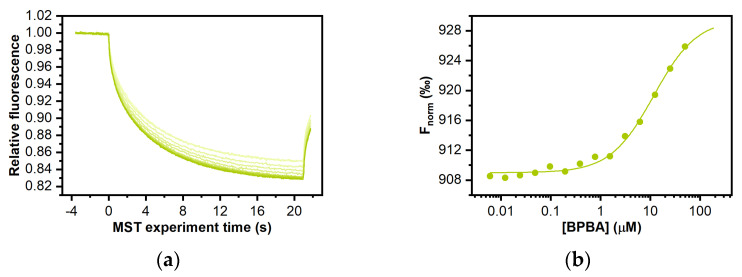
MST measurements on the interaction of compound **BPBA** with TERRA G4. (**a**) Time traces recorded by incubating increasing concentrations of **BPBA** with the labeled G4; (**b**) the corresponding binding curves.

To obtain quantitative data on the binding affinity of **BPBA** for the investigated G4s, and to confirm ligand selectivity for G4 over the duplex, microscale thermophoresis (MST) experiments were carried out. MST is a rapid and easy methodology to measure the affinity of a small molecule for a nucleic acid target in solution [80,81,82]. This technique is based on thermophoresis, the directed motion of molecules in small temperature gradients. Thermophoresis is highly sensitive to all types of binding-induced changes of molecular properties, be it in size, charge, hydration shell, or conformation. Thus, if the ligand binding to the investigated molecule alters at least one of these parameters, it also changes the thermophoretic behavior of the target. This effect can be used to evaluate equilibrium dissociation constant *K*_d_. To this purpose, serial dilutions of **BPBA** were prepared, mixed with a constant concentration of Cy5.5-labeled oligonucleotides (*TERRA G4*, *c-kit2 G4*, *c-myc G4*, *Tel*_23_ *G4*, or *Hrp*_20_), loaded into capillaries, and analyzed by MST. Results of MST binding curves (Figure 6 and Figure S7, Table 1) indicated that **BPBA** was able to bind to the parallel-stranded G4 structures, showing the lower *K*_d_ for *TERRA G4* (9.6 (±0.6) μM), followed by *c-kit2 G4* and *c-myc G4* (*K*_d_ = 23.5 (±0.6) μM and 47.5 (±0.4) μM, respectively) (Table 1). On the other hand, no significant change in the thermophoretic signal was observed for *Tel*_23_ *G4* and *Hrp*_20_ hairpin-duplex (Figure S7), clearly indicating the absence of a significant interaction in these cases.

These results agree with those obtained with other techniques and confirm the preferential binding of **BPBA** to parallel over antiparallel-stranded G4 topologies, and its selectivity for the G4 over duplex conformation. Additionally, although **BPBA** showed propensity to bind both *TERRA G4* and the parallel G4s *c-myc G4* and *c-kit2 G4*, it showed higher binding affinity for TERRA.

### 2.7. Antiproliferative Effect of BPBA in Low vs. High TERRA-Expressing Human Cancer Cells

**Figure 7:**
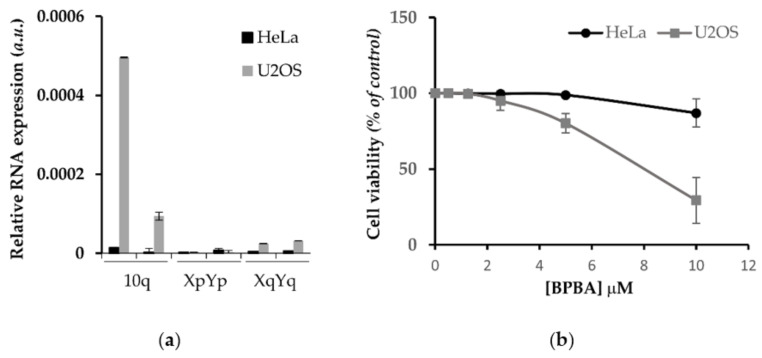
Differential effect of **BPBA** on viability of high vs. low TERRA expressing cells. (**a**) RT qPCR showing relative expression of specific TERRA RNA at different subtelomeric loci (10q, XpYp, XqYq) of HeLa, and U2OS cells; (**b**) HeLa and U2OS exposed to indicated doses of **BPBA** and, after 6 days, analyzed by crystal violet assay to determine the fraction of surviving cells. Percentages of surviving cells relative to untreated samples and mean of three independent experiments are shown. Error bars are SD.

G4 binders have an established antiproliferative effect in cancer cells depending on their ability to induce DNA damage response (DDR) or to inhibit the expression of cellular oncogenes. Since **BPBA** showed a high affinity to *TERRA G4*, we assessed the antiproliferative effect of this compound in correlation with TERRA expression. To this aim, we employed human cervix cancer cells (HeLa) characterized by telomerase activity and low TERRA expression, as well as human osteosarcoma cells (U2OS) lacking telomerase activity and expressing high levels of TERRA [83,84]. In those cell lines, TERRA expression was measured by RT qPCR assay with primers against some of the most active TERRA promoters located at subtelomeres of chromosomes 10q, XqYq, and XpYp, showing a huge difference of expression between the two cell lines (Figure 7a). Then, the viability of cells exposed to **BPBA** concentration ranging from 50 nM to 10 µM was assessed by crystal violet assay. As shown in Figure 7b, U2OS cells were significantly more sensitive to **BPBA** (IC_50_ = 8.1 (±1.0) µM) with respect to HeLa (IC_50_ >> 10 µM).

### 2.8. BPBA Stabilizes TERRA Levels in U2OS Cells

**Figure 8:**
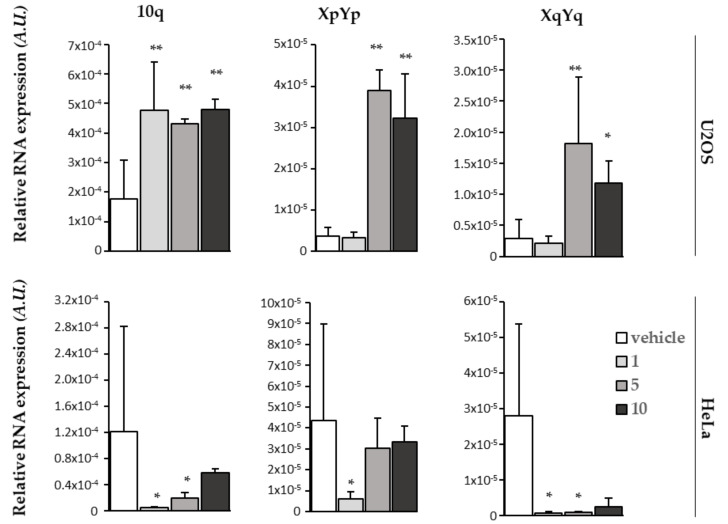
Effect of **BPBA** on TERRA expression. RT qPCR showing relative expression of specific TERRA RNA at different subtelomeric loci of HeLa and U2OS cells treated with the vehicle or indicated doses (1, 5, or 10 µM) of **BPBA** for 72 h. Mean of three independent experiments is shown. Error bars are SD; * = *p* < 0.5; ** = *p* < 0.1.

To gain insight into the mechanism underlying the differential biological effect of **BPBA** on high vs. low TERRA-expressing cells, we assessed the ability of **BPBA** to bind and stabilize TERRA in cellulo by RT qPCR analysis of relative TERRA expression upon treatment. To this aim, both HeLa and U2OS cell lines were exposed to different concentrations of **BPBA**; after 72 h, RNA was extracted and processed for TERRA analysis. Relative TERRA expression reported in Figure 8 clearly shows that **BPBA** induced a stabilization of TERRA expression in U2OS that led to an accumulation of the RNA within the cell. This effect reached a saturation point at 5 µM, when presumably all TERRA molecules were bound by the ligand and sequestered from the degradation complexes binding that regulate the physiological turnover of the lncRNA. We did not observe the same effect in HeLa, depending on the low abundance of TERRA.

### 2.9. BPBA Induces Persistent DDR Activation in U2OS Cells

**Figure 9:**
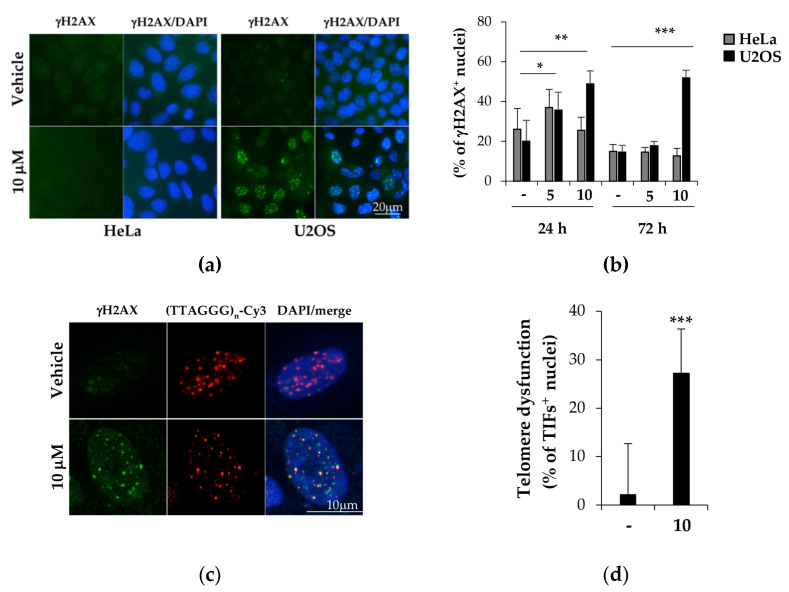
TERRA binding by **BPBA** triggers persistent DDR and telomere dysfunction in U2OS cells. HeLa and U2OS cells treated with indicated doses of **BPBA**. At each endpoint, cells were fixed and processed for immunofluorescence with the antiphosphorylated γH2AX mAb primary antibody, followed by goat–antimouse 488 secondary antibody (**a**,**b**) alone or (**c**,**d**) in combination with FISH using the telomere-specific (TTAGGG)_n_-Cy3 PNA probe. Fluorescence signals acquired with a Leica DMIRE deconvolution microscope (representative images at 72 h shown in (**a**)) or a Zeiss LMS confocal scanner (**b**) at 63× magnification. Percentage of cells displaying γH2AX signals or TIFs (>4 (TTAGGG)_n_-Cy3/γH2AX colocalizing spots) was scored and reported in histograms ((**b**,**d**), respectively). Histograms report the mean of at least 6 different fields per sample (*n* > 150). Error bars are SD; * = *p* < 0.5; ** = *p* < 0.1; *** = *p* < 0.05.

TERRA displacement from telomeres is supposed to induce telomere dysfunction [85]. To better understand the mechanisms underlying the cytotoxicity of **BPBA** in U2OS cells, we investigated the possibility that TERRA stabilization by **BPBA** could induce a DDR at telomeres (a marker for telomere dysfunction) leading to cell death. To this aim, both U2OS and HeLa cells were exposed to the compound for 24 or 72 h, and DDR activation was measured as the percentage of cells displaying γH2AX histone phosphorylation, a marker of DDR. As shown in Figure 9, HeLa treated with 5 or 10 µM **BPBA** showed a negligible DDR induction both in the first 24 h of treatment and in the following 72 h. Conversely, compared to the control, both 5 and 10 µM **BPBA** were able to induce DDR in a significant fraction of U2OS cells in the first 24 h (Figure 9b). However, while the 5 µM dose-induced DDR was rescued in the following 72 h, DDR was persistent at 72 h in 50% of the U2OS cells treated with 10 µM **BPBA**, in agreement with the calculated IC_50_ dose (Figure 7b). Lastly, to ascertain if the activated DDR coincided with telomeric loci, telomere-dysfunction-induced foci (TIF) activation was measured in U2OS cells treated with 10 µM **BPBA** for 72 h (Figure 9c). TIF positive cells (defined as cells displaying at least 4 of Cy3-conjugated telomere PNA probe/phosphorylated γH2AX histone colocalizing spots) were significantly increased in treated samples compared to in the control (Figure 9d).

## 3. Materials and Methods

### 3.1. Materials

CPG supports, (2’-OTBDMS)-RNA and DNA phosphoramidites, and all reagents for oligonucleotide synthesis were purchased from Link Technologies (Bellshill, UK). All other reagents and solvents were from Sigma-Aldrich (Merck KGaA, Darmstadt, Germany) and used without further purification. All buffers were produced from highly purified Milli-Q water and sterilized before use with diethylpirocarbonate (DEPC, from Merck KGaA, Darmstadt, Germany) and/or autocleavage. Putative ligands were purchased from Mcule (mcule.com Kft. Budapest, Hungary).

### 3.2. Virtual Screening

The 3D coordinates of the *TERRA G4* structures formed by the r(UAGGGUUAGGGU) sequence determined by NMR (PDB code: 2KBP) [54] and by X-ray diffraction (PDB code: 3IBK) [50] were downloaded from the Protein Data Bank website. PDB structures were prepared for docking using AutoDockTools by retaining nonstandard residues. Co-crystallized water molecules and counterions were removed from the X-ray structure.

A set of small organic solvent molecules, used as probes for hot spot mapping the binding surface of G4s [56], were gathered and prepared for docking in Mcule. Solvent molecules were docked to each target by using the Docking workflow step in Mcule (exhaustiveness: 8), which utilizes the Vina docking algorithm [55]. Binding sites were defined as cubes with 100 Å length in each direction to ensure that poses were evaluated on the surface of the whole RNA structures. Potential hot spots were located where the top binding pose of at least three different solvent molecules were overlapping. The centers of the hot spots were determined as the average of the X, Y, and Z coordinates of all atoms of the overlapping solvent molecules.

To analyze whether the proximity of the identified hot spots (hot spot regions) could be targeted by larger but still small molecules, a diverse set of 300 compounds were selected from the Mcule database containing 5.2 M compounds at the time of the selection. The selection included the following property filters: 150 ≤ mass ≤ 300; 0 ≤ logP ≤ 3; H-bond acceptors ≤ 3; H-bond donors ≤ 3; rings ≥ 1; rotatable bonds ≤ 3; heavy atoms ≥ 15. Rapid elimination of swill (REOS) [86] filter was also applied to eliminate compounds containing unwanted motifs. Lastly, the ‘diversity selection’ workflow step was used to select the most diverse (dissimilar) molecules by eliminating the closest analogs, thus maximizing the coverage of the chemical space. The resulting 300 compounds were docked by the Docking Vina workflow step in Mcule to binding sites defined around the center of each of the previously identified hot spots (cubes, length in each direction: 22 Å). Docking scores of the compounds were analyzed for each hot spot region.

Then, a diverse set of commercially available compounds was prepared as a screening library. We started our selection from the Mcule database containing millions of purchasable compounds. The following property filters were applied: mass ≥ 200 Da; logP ≥ 0; rule-of-five violations = 0; rings ≥ 1; rotatable bonds ≤ 4; heavy atoms ≥ 15. Subsequently, the REOS filter was applied to eliminate compounds with toxic or non drug-like substructures [86]. Lastly, a diverse set of a maximum of 0.7 Tanimoto coefficient was created on the basis of the OpenBabel linear fingerprint. These filters resulted in a screening library of 58870 compounds. The most relevant protonation state of these compounds at pH 7.4 was generated by OpenBabel 2.3.1. The generated screening library was docked into each identified binding site of each RNA structure. Calculations were run on Mcule using the Docking Vina workflow step with default settings. All compounds were ranked on the basis of their docking score. The distribution of the docking scores for each virtual screening was analyzed to ensure that the docking method could distinguish between different ligands, i.e., the scores of the top hits significantly differ from those at the end of the ranked screening database. After this step, 103 compounds were selected and purchased for further analysis. Stock solutions of these compounds were prepared at 10 mM in DMSO. No solubility problems were encountered for the putative ligands at any of the concentrations used in the various experiments.

### 3.3. Oligonucleotide Synthesis and Sample Preparation

RNA/DNA sequences were synthesized on an ABI 394 DNA/RNA synthesizer (Applied Biosystem, Foster City, CA, USA) using standard β-cyanoethyl phosphoramidite solid phase chemistry at 1 μmol synthesis scale. Regarding RNA synthesis, 5-benzylthio-1-H-tetrazole (BTT) instead of 4,5-dicyanoimidazole (DCI) was used as activator reagent, and coupling steps were prolonged of 5 min. Another difference concerned the deprotection of bases and phosphates. A concentrated NH_4_OH/EtOH (3:1, *v*/*v*) solution was used in the case of RNA, and the reaction was left at r.t. for 12 h. For DNA sequences, deprotection and detachment were performed by using a concentrated NH_4_OH aqueous solution at 55 °C for 12 h. Both RNA and DNA sequences were purified by high-performance liquid chromatography (HPLC) on a Nucleogel SAX column (1000-8/46, Macherey-Nagel, GmbH & Co. KG, Dueren, Germany), as previously reported [87]. The fractions of the oligomers were collected and successively desalted by Sep-Pak cartridges (C-18). Lastly, 2′-TBDMS groups in RNA were removed by Et_3_N·3HF/DMF (1:3, *v*/*v*) at r.t. for 12 h. The reaction was quenched with 0.1 M TEAA buffer (pH 7.0) and again desalted on a Sep-pak (C-18) cartridge. All oligonucleotides were proven to be >98% pure by NMR. The following oligonucleotides were synthesized: the 12-mer truncation of the human telomeric repeat-containing RNA sequence r(UAGGGUAAGGGU) (*TERRA G4*); the G4-forming sequence from GSEC long noncoding RNA r(GGGGUGGAGGAGGGGGAAGGGCGGGGG) (*GSEC G4*); the G-rich sequence of the 5′-UTR of Bcl-2 mRNA (GGGCCGUGGGGUGGGAGCUGGG) (*Bcl-2 G4*); the *c-Kit2* sequence from the *c-Kit* oncogene promoter d(CGGGCGGGCGCTAGGGAGGGT) (*c-kit2 G4*); *c-Myc* promoter sequence d(TGAGGGTGGGTAGGGTGGGTAA) (*c-myc G4*); the 23-mer truncation of the human telomeric sequence d(TAGGGTTAGGGTTAGGGTTAGGG) (*Tel*_23_ *G4*); the 20-mer hairpin duplex-forming sequence d(CGAATTCGTTTTCGAATTCG) (*Hrp*_20_); and the 27-mer hairpin duplex-forming sequence d(CGCGAATTCGCGTTTCGCGAATTCGCG) (*Hrp*_27_). Oligonucleotides were prepared in the appropriate buffer, and their concentration was measured by UV adsorption at 90 °C using the appropriate molar extinction coefficient values, ε (λ = 260 nm), calculated by the nearest-neighbor model [88].

### 3.4. Circular Dichroism (CD) Experiments

CD experiments were performed on a Jasco J-815 spectropolarimeter (Jasco, Easton, MD, USA) equipped with a PTC-423S/15 Peltier temperature controller. All spectra were recorded at 20 and 100 °C in the wavelength range of 230–320 nm, and averaged over three scans. A scan rate of 100 nm/min with a 1 s response time and 1 nm bandwidth were used. The buffer baseline was subtracted from each spectrum. Concentrations of 2 μM for G4s and 4 μM for *Hrp*_20_ were used. RNA G4s were prepared in 20 mM KH_2_PO_4_/K_2_HPO_4_ buffer (pH 7.0) containing 70 mM KCl, while a buffer solution consisting of 5 mM KH_2_PO_4_/K_2_HPO_4_ (pH 7.0) containing 20 mM KCl was used for all DNA samples. All oligonucleotide samples were annealed by heating at 90 °C for 5 min, followed by a slow cooling to room temperature overnight. CD spectra of oligonucleotide/ligand mixtures were obtained by adding 10 molar equiv of ligand (stock solutions of ligands were 10 mM in DMSO). CD melting experiments were carried out in the 20–100 °C temperature range at a 1 °C/min heating rate by following changes of the CD signal at the wavelengths of the maximal CD intensity (i.e., 264 nm for *TERRA G4*, *GSEC G4*, *Bcl-2 G4*, *c-kit2 G4*, and *c-myc G4*; 287 nm for *Tel*_23_ *G4*; 280 nm for *Hrp*_20_). CD melting experiments were performed in the absence and presence of ligands (10 molar equiv) added to the folded nucleic acid structures. The apparent melting temperatures (*T*_m_) were determined from a curve fit using Origin 7.0 software (OriginLab Corp., MA, USA). Δ*T*_m_ values were determined as the difference in the *T*_m_ values of the nucleic acid structures in the presence and absence of ligands. All experiments were performed in triplicate, and the reported values are the average of the three measurements.

### 3.5. FRET Melting Experiments

Measurements were carried out on a FP-8300 spectrofluorometer (Jasco, Easton, MD, USA) equipped with a Peltier temperature controller system (Jasco PCT-818) using the dual-labeled G4-forming telomeric RNA sequence FAM-[r(GGGUAAGGGUAAGGGUAAGGG)]-TAMRA (*F-TERRA-T*), provided from Biomers (Ulm, Germany). The oligonucleotide was dissolved in water at 1 mM, diluted at 1 μM using 5 mM potassium phosphate buffer (pH 7.0) containing 20 mM KCl, and lastly annealed by heating to 90 °C for 5 min, followed by cooling to room temperature overnight and storage at 4 °C for 24 h before data acquisition. Experiments were performed in sealed quartz cuvettes with a path length of 1 cm by using 0.2 μM of prefolded *F-TERRA-T* G4 target, the ligand at 2 μM, and the *Hrp*_27_ duplex competitor at 0, 6, and 20 μM final concentrations [61,62,63,64]. In addition, a blank with no compound or competitor was also analyzed. Fluorescence spectra were acquired before (at 15 °C) and after (at 90 °C) melting assay. The dual-labeled oligonucleotide was excited at 492 nm, and emission spectra were recorded between 500 and 650 nm by using 100 nm/s scan speed. Excitation and emission slit widths were both set at 5 nm. FRET melting was performed by monitoring the emission of FAM at 522 nm (upon excitation at 492 nm), using a heating gradient of 1 °C/min over the range 15–90 °C. Emission of FAM was normalized between 0 and 1. Final analysis of the data was carried out using Origin 7.0 software (OriginLab Corp., MA, USA).

### 3.6. Fluorescent Intercalator Displacement (FID) Assay with Thiazole Orange (TO)

A solution containing 0.25 μM of prefolded RNA (*TERRA G4*) or DNA G4 target and 0.5 μM of TO in 20 mM KH_2_PO_4_ (pH 7.0) and 70 mM KCl was prepared in a 1 cm path-length cell, and the corresponding fluorescence spectrum was acquired in the absence and presence of increasing concentrations of **BPBA** (10 mM stock solution in pure DMSO). Each ligand addition (from 0.5 to 20 molar equiv) was followed by a 3 min equilibration time before spectrum acquisition. The FID experiment was extended to a duplex DNA model (*Hrp*_27_), in this case, three equivalents of TO (0.75 μM) were added to an oligonucleotide solution (0.25 μM). Measurements were run at 20 °C on a FP-8300 spectrofluorometer (Jasco, Easton, MD, USA) equipped with a Peltier cell holder (Jasco PCT-818), using an excitation wavelength of 485 nm and recording the emission in the 500–650 nm wavelength range. Both excitation and emission slits were set at 5 nm. The percentage of TO displacement was calculated as TO displacement (%) = 100 − [(F/ F_0_) × 100], where F_0_ is the fluorescence in the absence of ligand and F the fluorescence after each ligand addition. The percentage of displacement was then plotted as a function of the ligand concentration, and DC_50_ was calculated as the required concentration to displace 50% TO from each investigated DNA. Each titration was repeated at least in triplicate.

### 3.7. Fluorescent Intercalator Displacement (FID) Assay with Ethidium Bromide (EB)

A solution containing 10 μM of prefolded *TERRA G4* and 5 μM of EB in 20 mM potassium buffer (pH 7.0) containing 70 mM KCl was prepared in a 1 cm path-length cell. The fluorescence spectrum of the EB/*TERRA G4* complex in the absence of ligand was first recorded. Then, increasing concentrations of **BPBA** (10 mM stock solution in pure DMSO) were mixed to this EB/*TERRA G4* complex, and spectra were recorded 3 min after each ligand addition. Measurements were run at 20 °C on a FP-8300 spectrofluorometer (Jasco, Easton, MD, USA) equipped with a Peltier cell holder (Jasco PCT-818), using an excitation wavelength of 510 nm, and recording the emission in the 550–700 nm wavelength range. Both excitation and emission slits were set at 5 nm. Experiments were performed in duplicate.

### 3.8. MicroScale Thermophoresis (MST) Experiments

MST measurements were performed on a Monolith NT.115 (Nanotemper Technologies, Munich, Germany). The Cy5.5-fluorescently labelled oligonucleotides (from Biomers, Ulm, Germany) were prepared at 10–20 µM in 20 mM KH_2_PO_4_ buffer (pH 7.0) containing 70 mM KCl and annealed as described above. Nucleic acid samples were then diluted using the same phosphate buffer supplemented with 0.1% Tween. Ligand stock solution was 2 mM in pure DMSO. For the MST experiments, the concentration of the labelled G4s was kept constant at 50 nM, while a serial dilution of the ligand (1:2 from 0.4 mM) in the same buffer supplemented with 20% DMSO, was prepared and mixed with the oligonucleotide solution with a volume ratio of 1:1. All samples containing 10% DMSO as the final concentration were loaded into standard capillaries (NanoTemper Technologies, Munich, Germany). Measurements were performed at 20 °C using autotune LED power and medium MST power. MST data analysis was performed by employing the MO. Affinity Analysis software (v2.3) provided with the instrument.

### 3.9. Cells and Viability Assay (Crystal Violet)

HeLa and U2OS cells were purchased from ATCC and maintained according to the purchaser’s instructions. Cells were seeded in 24 wells; after 24 h, cells were exposed to **BPBA** concentrations ranging from 1.25 to 10 µM for 6 days. Then, cells were washed twice in DPBS and fixed with 4% formaldehyde for 15 min at r.t. After washing with DPBS, 300 μL of crystal violet staining solution (Sigma-Aldrich, St. Louis, MO, USA) was added to each well and incubated for 30 min at r.t. Lastly, plates were rinsed twice with water, air-dried at r.t., and cell pellets were dissolved in 400 μL of acetic acid. The optical density of each well in triplicate was measured at 570 nm (OD_570_) with a 96-well plate in an ELISA reader (Falcon, Corning, NY, USA). The average absorbance in each condition was used to calculate the survival expressed as percent of treated vs. untreated condition. IC_50_ (the necessary dose to reduce survival of 50%) was calculated by Calcusyn software (Biosoft, Cambridge, UK).

### 3.10. Immunofluorescence/FISH

HeLa and U2OS cells were seeded and treated with **BPBA**. At each endpoint, cells were fixed in 2% formaldehyde, permeabilized in 0.25% Triton X-100 in PBS for 5 min at r.t., and incubated with the mouse mAb anti-γH2AX (Millipore, Burlington, MA, USA) followed by the secondary Alexa 488 goat antimouse antibody. Lastly, nuclei were counterstained with DAPI (Sigma-Aldrich, St. Louis, MO, USA). For combined FISH experiments, after immunofluorescence, samples were refixed in 2% formaldehyde and dehydrated by ethanol series. Then, slices were hybridized with telomere-specific (TTAGGG)_n_-Cy3 PNA probe (Panagene, Daejeon, South Korea) according to the manufacturer’s instruction. Lastly, samples were counterstained with DAPI (Sigma-Aldrich, St. Louis, MO, USA). Fluorescence signals were recorded by using a Leica DMIRE2 microscope equipped with a Leica DFC 350FX camera and elaborated by a Leica FW4000 deconvolution software (Leica, Solms, Germany) at 63× magnification. TIFs images were acquired with a Zeiss LSM confocal laser scanner (Zeiss, Jena, Germany) at 63× magnification.

### 3.11. TERRA Real-Time qPCR

Real-time qPCR analysis of TERRA was performed as described [89]. Briefly, RNA was extracted from cells with an RNAeasy mini kit (Quiagen, Hilden, Germany) and accurately digested with the RNase-free DNase set (Quiagen, Hilden, Germany). Then, RNA quality was checked on FA gel electrophoresis and amplified in real-time PCR assay with subtelomere specific primers with a 7900HT Fast Real Time PCR System (Applied Biosystem, Waltham, MA, USA).

## 4. Conclusions

Targeting noncanonical nucleic acid structures such as G4s is an appealing opportunity for drug intervention in anticancer therapy. Indeed, these unusual arrangements, and in particular their structural conversions, appear to play roles in regulating some important disease-related biological processes. Low-molecular-weight compounds affecting nucleic acid conformational equilibria by preferentially binding to a given form could, therefore, represent a real chance for therapeutic applications. Besides regulating telomerase activity and protecting chromosome ends from telomere degradation, G4-forming TERRA RNA also takes part in heterochromatin formation and homologous recombination, thus representing a valuable therapeutic target. Herein, the application of a virtual screening approach in tandem with experimental screening via CD melting assay succeeded in the identification of a new hit compound (**BPBA**) as a binder of TERRA G4. The in vitro G4 binding properties of this compound were characterized by several biophysical assays (CD, FRET, FID, and MST). CD and FRET melting assays, as well as MST experiments, revealed that **BPBA** features high selectivity toward G4s, being its binding to duplex DNA negligible. Furthermore, TO-FID and MST experiments showed that **BPBA** has enhanced binding affinity towards TERRA G4 vs. other G4-forming DNA sequences present along the human genome. The examination of the molecular structure of **BPBA** compared to other screened compounds suggests that its preferable binding properties may be due to the presence of two benzoimidazole units connected by an aniline residue. This molecular arrangement, which is present only in this compound, gives the molecule extensive planarity, and probably also allows for the optimal distribution of polar groups for interaction with TERRA G4.

Biological characterization demonstrated that **BPBA** can bind and stabilize in cellulo TERRA lncRNAs, probably by sequestering them from the physiological turnover cell machinery. Moreover, TERRA stabilization induced a DDR, putatively by displacing TERRA from telomeric DNA. Indeed, TERRA physically interacts with telomeric chromatin by forming DNA:RNA hybrids that are required for telomere homeostasis, especially in ALT cells such as U2OS, where TERRA downregulation causes the formation of TIFs [85]. In agreement with this, DDR activation prevalently occurs in U2OS cells, where **BPBA** also has the highest cytotoxic effect.

Overall, this study demonstrates that it is possible to identify TERRA G4 binders with potential pharmacological effects, thus paving the way for the search of new RNA-targeting drug candidates. A relevant percentage of human tumors (around 15%) possess ALT mechanisms for telomere elongation that correlate with high TERRA expression. These tumors are prevalently of mesenchymal origin. They are characterized by high genetic instability, and, in many histotypes, ALT positivity is associated with worse prognosis [90]. In this regard, TERRA G4 ligands could represent an effective pharmacological strategy to hit this class of tumors.

## Figures and Tables

**Table 1:** Analysis of **BPBA** interaction with the investigated oligonucleotides.

**∆*T*_m_ (°C) ^1^**	**Circular Dichroism (CD) Melting**									
	*TERRA G4*	*c-kit2 G4*		*c-myc G4*		*Tel*_23_ *G4*	*GSEC G4*		*Bcl-2 G4*	*Hrp*_20_
	4.5 (±0.4)	18.7 (±0.3)		9.4 (±0.3)		0.7 (±0.2)	1.7 (±0.3)		2.9 (±0.3)	1.0 (±0.5)
	**Förster resonance energy transfer (FRET) melting**									
	*F-TERRA-T*				*F-TERRA-T + Hrp*_27_ (1:30)			*F-TERRA-T + Hrp*_27_ (1:100)		
	3.6 (±0.2)				3.7 (±0.2)			3.1 (±0.2)		
**DC_50_ (µM)**	**Fluorescent intercalator displacement (FID)—thiazole orange**									
	*TERRA G4*		*c-kit2 G4*			*c-myc G4*		*Tel*_23_ *G4*		*Hrp*_27_
	2.4 (±0.4)		3.8 (±0.6)			n.d. ^2^		n.d. ^2^		4.1 (±0.5)
	**Fluorescent intercalator displacement (FID)—ethidium bromide**									
	*TERRA G4*									
	1.3 (±0.4)									
***K*_d_ (µM)**	**Microscale thermophoresis (MST)**									
	*TERRA G4*		*c-kit2 G4*			*c-myc G4*		*Tel*_23_ *G4*		*Hrp*_20_
	9.6 (±0.6)		23.5 (±0.6)			47.5 (±0.4)		n.d. ^2^		n.d. ^2^

^1^ Δ*T*_m_ = *T*_m (oligonucleotide+10 ligand equiv)_ − *T*_m (oligonucleotide)_. *T*_m_ values in the absence of ligand are *TERRA G4* = 74.3 (±0.1) °C; *c-kit2 G4* = 59.7 (±0.1) °C; *c-myc G4* = 75.5 (±0.1) °C; *Tel*_23_ *G4* = 53.7 (±0.1) °C; *GSEC G4* = 78.8 (±0.1) °C; *Bcl-2 G4* = 74.7 (±0.2) °C; *Hrp*_20_ = 65.5 (±0.2) °C; *F-TERRA-T* = 73.5 (±0.1) °C. All experiments were performed in duplicate, and reported values are the average of two measurements. ^2^ n.d. = not determinable.

## Data Availability

Not applicable.

## Supplementary Materials

The following are available online at https://www.mdpi.com/article/10.3390/ijms221910315/s1.

## Abbreviations

ALT	Alternative lengthening of telomeres
CD	Circular dichroism
DDR	DNA damage response
EB	Ethidium bromide
FID	Fluorescent intercalator displacement
FISH	Fluorescence in situ hybridization
FRET	Förster resonance energy transfer
G4	G-quadruplex
LncRNAs	Long noncoding RNAs
MST	Microscale thermophoresis
TERRA	Telomere repeat containing RNA
TIF	Telomere dysfunction-induced foci
TO	Thiazole orange
