# Supplementary material for: Targeting of Telomeric Repeat-Containing RNA G-Quadruplexes: From Screening to Biophysical and Biological Characterization of a New Hit Compound
Source: Int J Mol Sci. 2021 Sep 24;22(19):10315. doi: 10.3390/ijms221910315 (PMC8508872; doi:10.3390/ijms221910315)
Supplement: Supplementary file 1 [file ijms-22-10315-s001.zip › ijms-1363645-supplementary.pdf]

Supplementary Material

Targeting of Telomeric Repeat-Containing RNA G-Quadruplexes: From Screening to Biophysical and Biological Characterization of a New Hit Compound

Simona Marzano [1], Bruno Pagano [1], Nunzia Iaccarino [1], Anna Di Porzio [1], Stefano De Tito [2,3], Eleonora Vertecchi [4], Erica Salvati [4], Antonio Randazzo [1] and Jussara Amato [1,*]

[1] Department of Pharmacy, University of Naples Federico II, Via D. Montesano 49, 80131 Naples, Italy; simona.marzano@unina.it (S.M.); bruno.pagano@unina.it (B.P.); nunzia.iaccarino@unina.it (N.I.); anna.diporzio@unina.it (A.D.P.); antonio.randazzo@unina.it (A.R.)

[2] Molecular Cell Biology of Autophagy, The Francis Crick Institute, 1 Midland Road, London NW1 1AT, UK; stefano.de-tito@crick.ac.uk

[3] Institute of Experimental Endocrinology and Oncology, National Research Council, 80131 Naples, Italy

[4] Institute of Molecular Biology and Pathology, National Research Council, c/o Department of Biology and Biotechnology "C. Darwin", Sapienza University of Rome, Via degli Apuli 4, 00185 Rome, Italy; eleonora.vertecchi@uniroma1.it (E.V.); erica.salvati@cnr.it (E.S.)

* Correspondence: jussara.amato@unina.it, Tel.: +39-081678630

**Table of contents**	**Page**
Table S1	S2
Figure S1	S17
Figure S2	S21
Figure S3	S25
Table S2	S25
Figure S4	S26
Figure S5	S27
Figure S6	S28
Figure S7	S29

Table S1. Vendor code, docking score, compound-induced thermal stabilization of *TERRA G4* measured by CD melting experiments, and chemical structure for each of the compounds studied.

Entry	Mcule ID	Docking score	ΔT_m (°C) [a]	Chemical structure
**1**	4200333985	- 9.0	+ 1.0 (± 0.3)	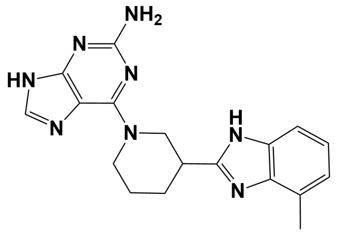
**2**	9535445570	- 8.3	+ 1.1 (± 0.3)	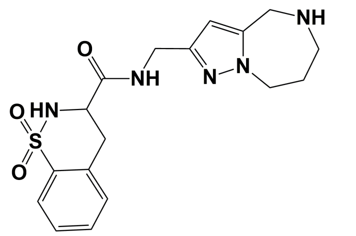
**3**	8125263327	- 9.0	+ 0.5 (± 0.3)	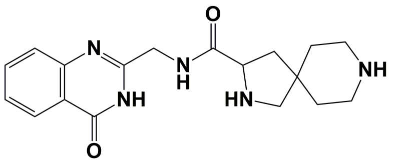
**4**	4870649872	- 7.6	+ 1.0 (± 0.3)	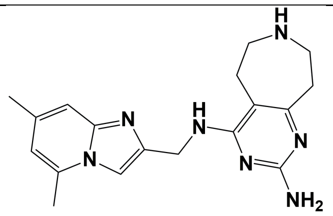
**5**	4039411788	- 9.2	+ 0.5 (± 0.2)	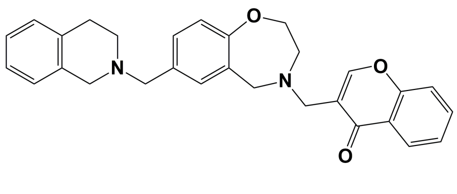
**6**	9122563985	-7.5	+ 0.7 (± 0.4)	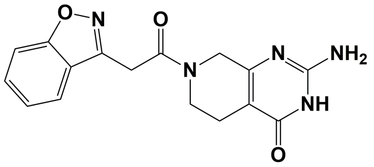
**7**	5832326880	- 8.8	0.0 (± 0.3)	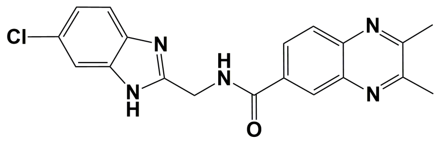
**8**	4149197811	- 7.9	+ 0.1 (± 0.3)	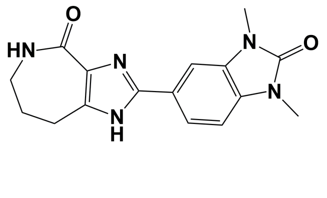

**9**	2371430517	- 8.7	+ 0.7 (± 0.2)	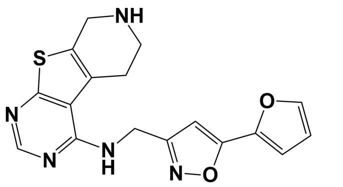
**10**	8943827427	- 8.2	- 0.4 (± 0.3)	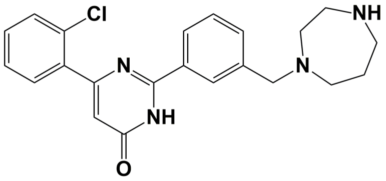
**11**	6518006383	- 7.4	+ 0.6 (± 0.3)	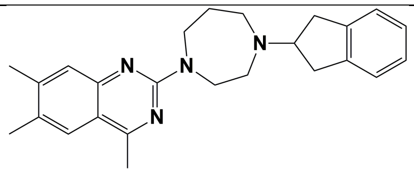
**12**	2821959251	- 8.0	+ 1.4 (± 0.3)	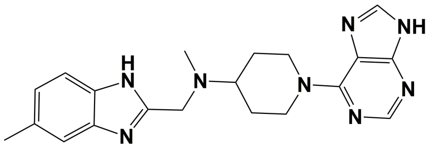
**13**	9820891813	- 8.6	+ 0.9 (± 0.3)	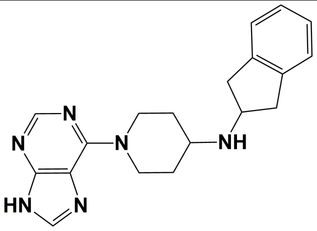
**14**	6646365388	- 8.7	+ 2.7 (± 0.3)	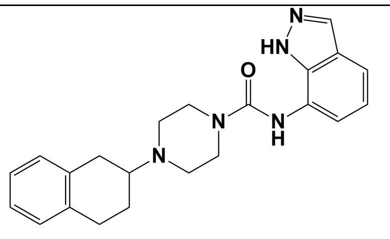
**15**	9000656427	- 9.1	+ 0.4 (± 0.3)	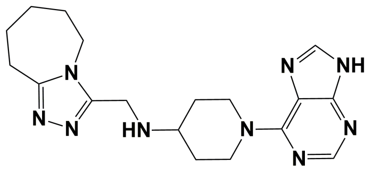
**16**	2237014444	- 7.9	+ 0.1 (± 0.2)	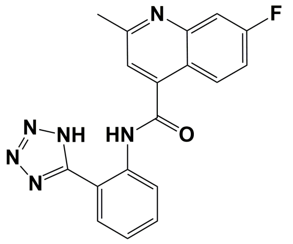

**17**	4686727035	- 8.0	+ 0.5 (± 0.3)	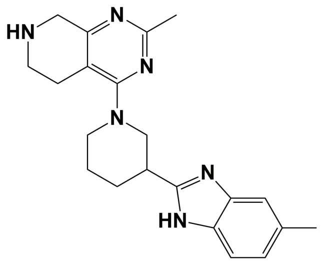
**18**	625202616	- 8.3	+ 1.3 (± 0.3)	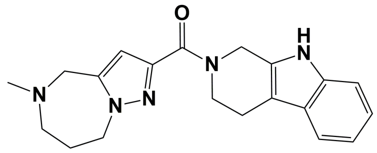
**19**	1582886421	- 7.7	+ 1.6 (± 0.3)	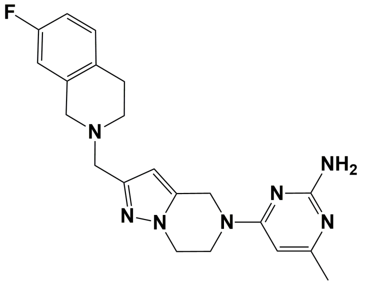
**20**	9309662623	- 8.8	+ 0.6 (± 0.2)	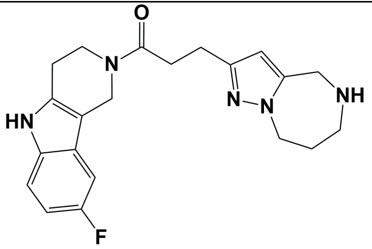
**21**	2256367138	- 8.1	+ 0.3 (± 0.3)	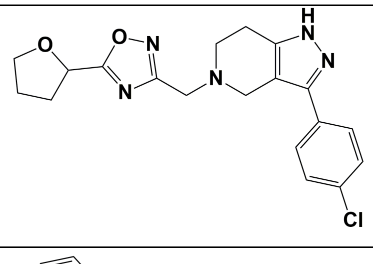
**22**	8544129449	- 7.4	+ 0.2 (± 0.2)	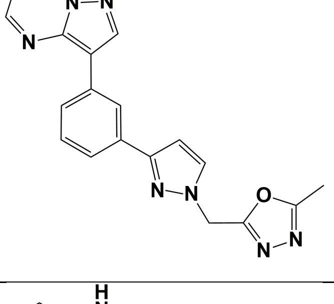
**23**	5271302873	- 8.7	+ 2.0 (± 0.2)	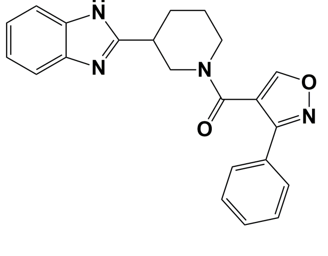

**24**	7012365698	- 8.1	- 0.4 (± 0.2)	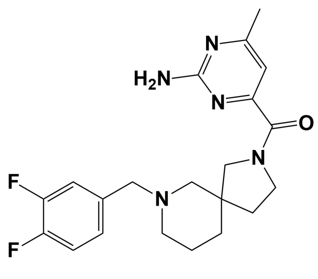
**25**	2689903110	- 7.6	+ 0.6 (± 0.2)	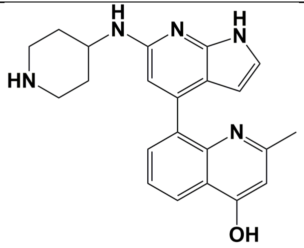
**26**	3536962472	- 8.0	- 1.0 (± 0.3)	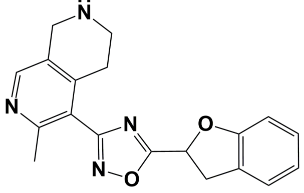
**27**	3956273338	- 7.8	+ 1.2 (± 0.3)	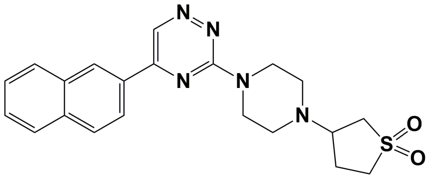
**28**	7883384392	- 8.0	+ 1.6 (± 0.3)	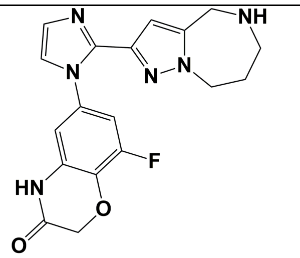
**29**	7861438091	- 8.1	+ 0.8 (± 0.3)	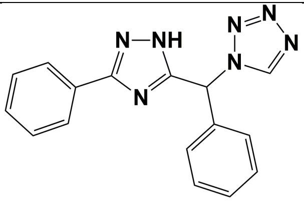
**30**	8863279206	- 7.5	+ 0.6 (± 0.3)	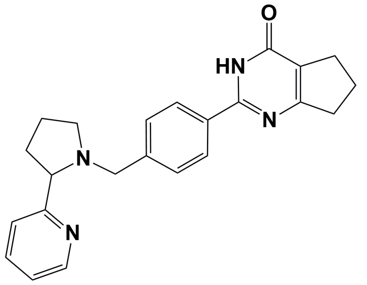

**31**	6951319952	- 8.2	- 1.6 (± 0.3)	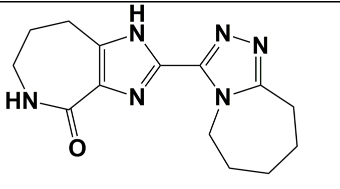
**32**	2790312956	- 8.1	+ 1.9 (± 0.3)	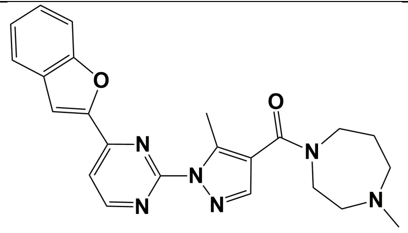
**33**	6218525125	- 7.5	+ 2.3 (± 0.3)	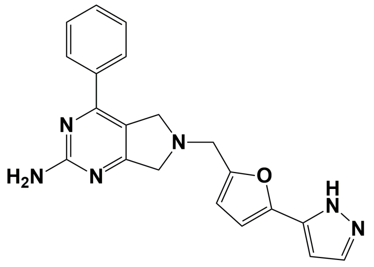
**34**	4430506216	- 8.0	+ 0.9 (± 0.2)	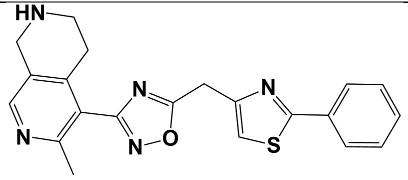
**35**	4354735787	- 9.4	+ 2.3 (± 0.3)	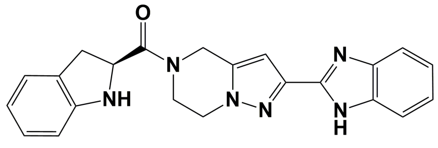
**36**	7408976836	- 7.9	+ 0.5 (± 0.2)	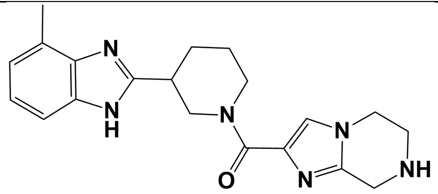
**37**	2094930759	- 7.7	+ 0.4 (± 0.2)	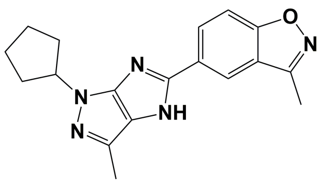
**38**	9572818472	- 8.0	+ 1.4 (± 0.3)	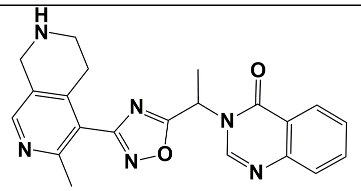

**39**	1485594099	- 7.9	- 0.8 (± 0.3)	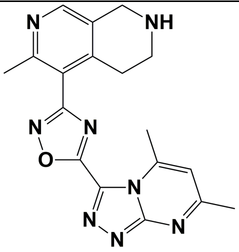
**40**	3974842418	- 8.0	- 0.4 (± 0.3)	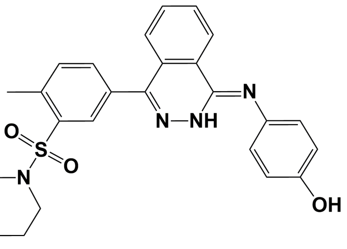
**41**	5970172123	- 7.8	+ 0.6 (± 0.3)	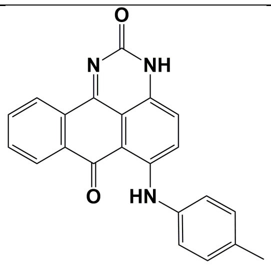
**42**	7100604422	- 7.5	+ 0.7 (± 0.2)	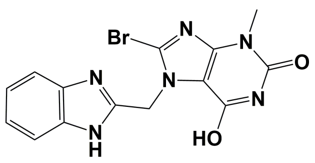
**43**	6521389889	- 7.7	+ 0.8 (± 0.2)	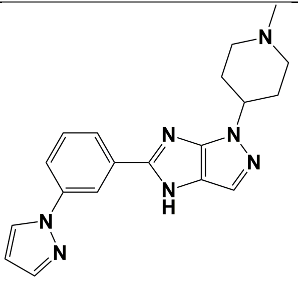
**44**	2338417829	- 9.0	+ 0.2 (± 0.3)	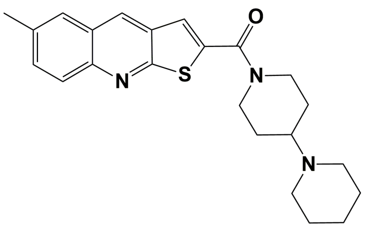
**45**	5477566010	- 7.6	+ 2.4 (± 0.4)	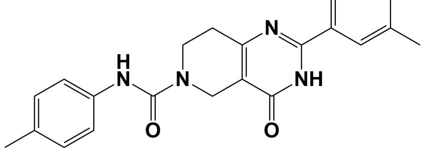

**46**	6462743924	- 8.2	+ 0.7 (± 0.2)	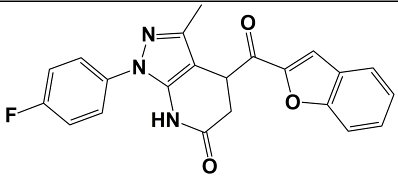
**47**	5356872927	- 9.0	+ 2.7 (± 0.2)	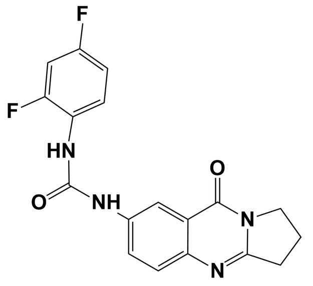
**48**	8744685790	- 7.6	+ 2.0 (± 0.6)	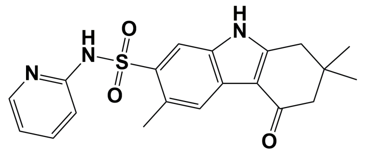
**49**	7664655383	- 7.8	+ 0.6 (± 0.4)	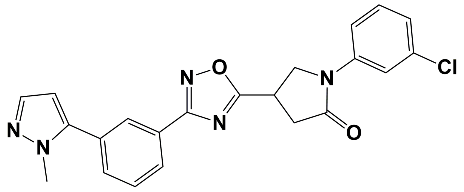
**50**	1713122274	- 7.4	- 0.2 (± 0.3)	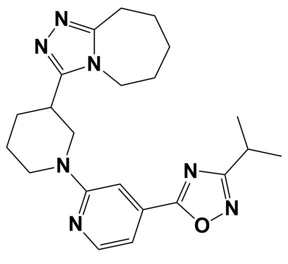
**51**	6509595317	- 8.0	+ 1.9 (± 0.5)	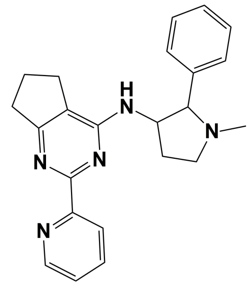
**52**	3540486896	- 8.6	+ 0.9 (± 0.3)	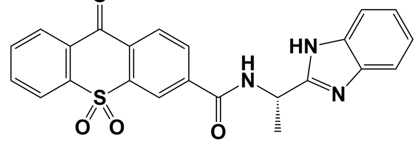

**53**	5658699201	- 8.2	- 1.3 (± 0.4)	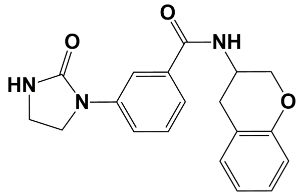
**54**	1711648004	- 7.5	- 0.7 (± 0.2)	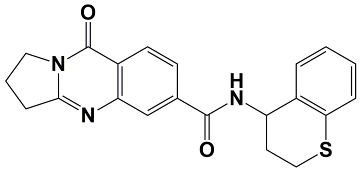
**55**	5257323893	- 8.1	+ 1.0 (± 0.2)	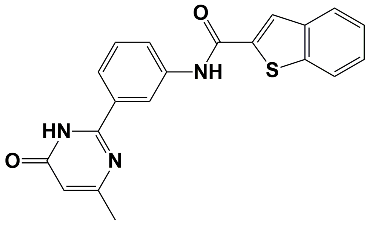
**56**	9383820746	- 8.2	+ 2.8 (± 0.3)	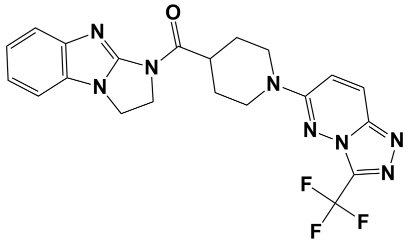
**57**	5467071397	- 8.2	- 0.3 (± 0.4)	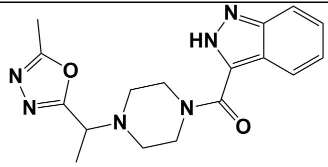
**58**	6995867112	- 7.4	+ 0.5 (± 0.2)	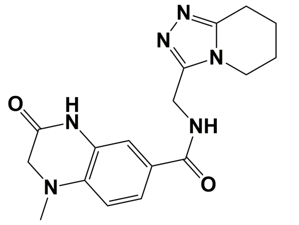
**59**	3244280719	- 8.7	+ 1.2 (± 0.5)	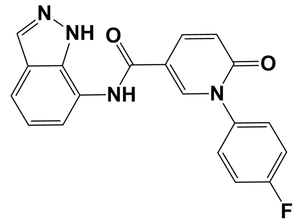
**60**	4322321290	- 7.4	- 0.4 (± 0.3)	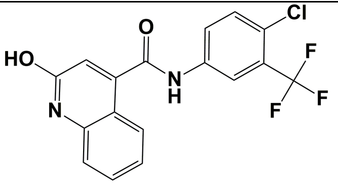

**61**	3496019129	- 7.6	+ 0.2 (± 0.6)	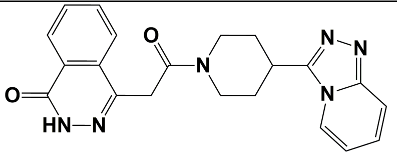
**62**	6106804983	- 8.2	- 3.1 (± 0.3)	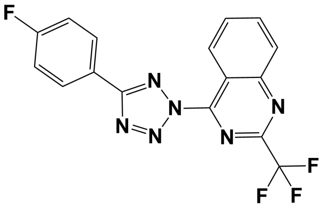
**63**	1016726876	- 7.5	- 0.4 (± 0.3)	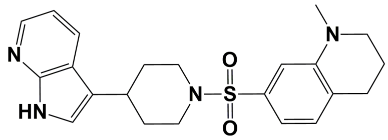
**64**	9257453767	- 8.3	+ 0.4 (± 0.2)	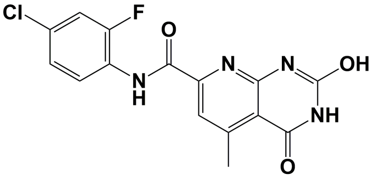
**65**	3603474871	- 7.4	+ 0.8 (± 0.3)	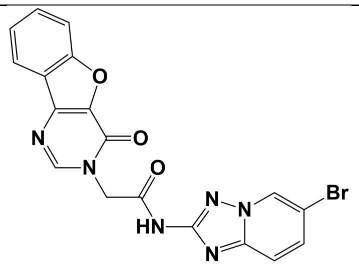
**66**	8644783008	- 8.2	+ 0.3 (± 0.3)	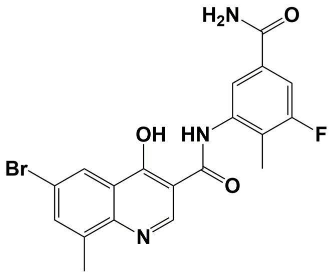
**67**	2087958632	- 8.0	- 0.8 (± 0.3)	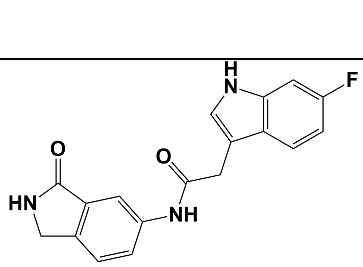

**68**	9565622799	- 8.1	+ 1.9 (± 0.5)	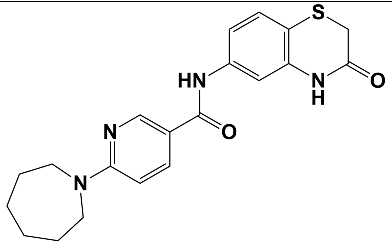
**69**	6247549013	- 9.2	+ 1.2 (± 0.2)	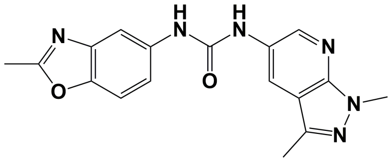
**70**	8704362400	- 8.1	+ 1.3 (± 0.3)	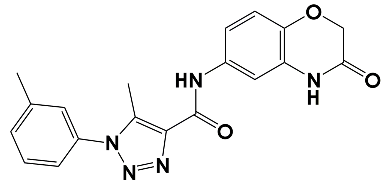
**71**	3259529912	- 8.0	+ 1.0 (± 0.2)	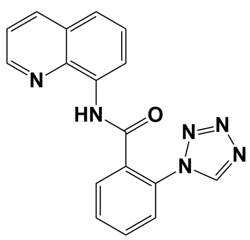
**72**	1157333157	- 8.0	+ 1.1 (± 0.2)	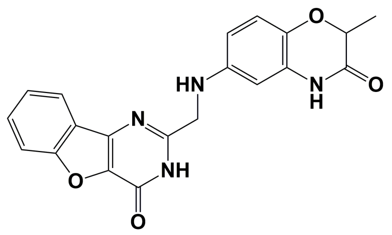
**73**	8958352416	- 8.0	+ 0.5 (± 0.3)	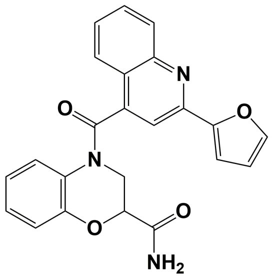
**74**	9240481846	- 8.4	+ 0.7 (± 0.3)	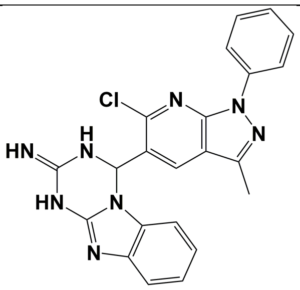

**75**	2792406159	- 9.0	+ 0.2 (± 0.4)	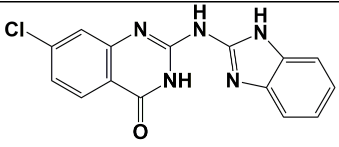
**76**	3698111653	- 9.0	+ 1.9 (± 0.2)	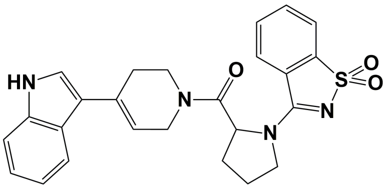
**77**	9503462548	- 8.3	+ 1.6 (± 0.3)	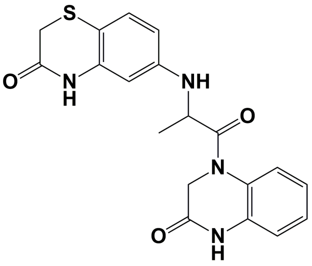
**78**	2899066240	- 8.0	0.0 (± 0.3)	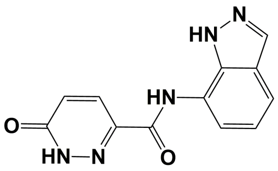
**79**	5286304768	- 7.9	+ 0.1 (± 0.2)	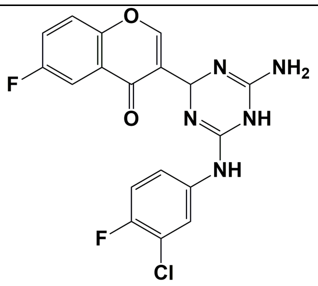
**80**	4220808496	- 8.1	+ 1.4 (± 0.4)	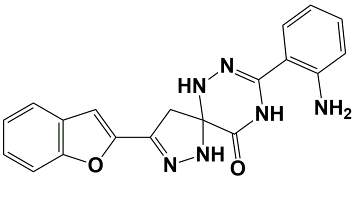
**81**	7136053894	- 7.4	+ 0.6 (± 0.6)	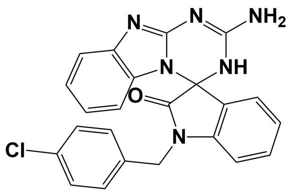

**82**	1075699151	- 7.5	- 0.6 (± 0.3)	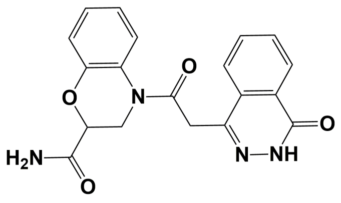
**83**	6350581691	- 7.5	- 0.8 (± 0.4)	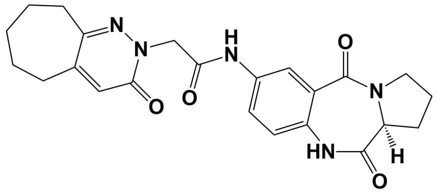
**84**	6567089130	- 9.3	- 0.3 (± 0.8)	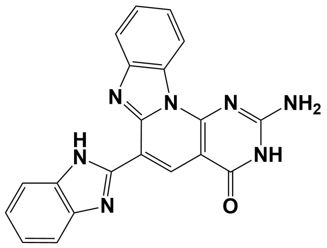
**85**	1079842730	- 9.2	+ 1.3 (± 0.4)	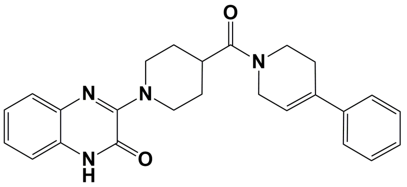
**86**	8335400599	- 7.4	+ 2.5 (± 0.3)	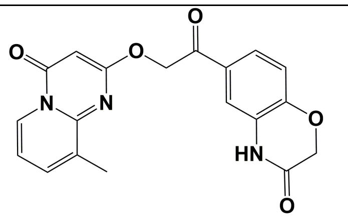
**87**	8543901493	- 8.2	+ 1.6 (± 0.2)	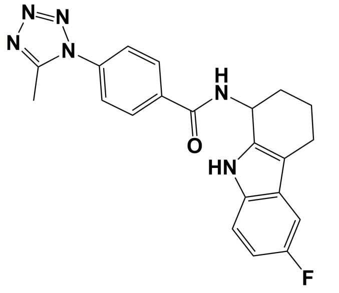
**88**	1940876099	- 8.2	+ 0.2 (± 0.5)	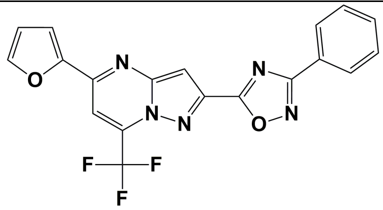

**89**	8948344198	- 8.0	- 0.8 (± 0.3)	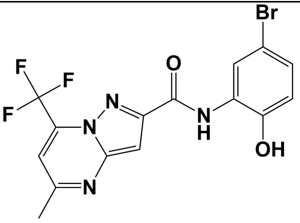
**90**	9965083627	- 7.8	- 1.4 (± 0.4)	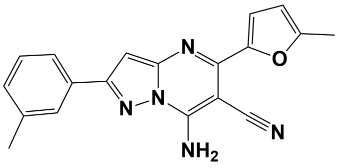
**91**	9253771008	- 8.0	+ 0.2 (± 0.2)	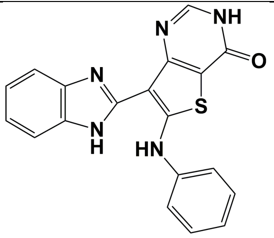
**92** (**BPBA**)	9923854130	- 9.8	+ 4.5 (± 0.4)	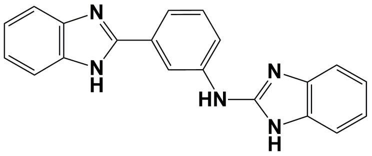
**93**	8688994319	- 7.7	+ 1.8 (± 0.3)	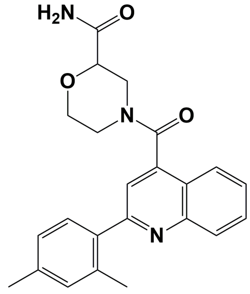
**94**	6114356071	- 8.1	- 0.5 (± 0.2)	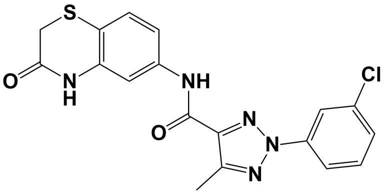
**95**	9584498449	- 8.1	+ 1.4 (± 0.2)	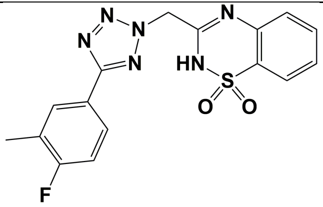

**96**	7696307933	- 8.9	+ 2.6 (± 0.9)	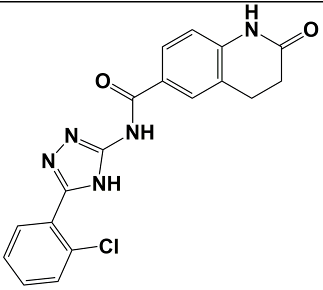
**97**	1317099783	- 7.5	+ 0.9 (± 0.2)	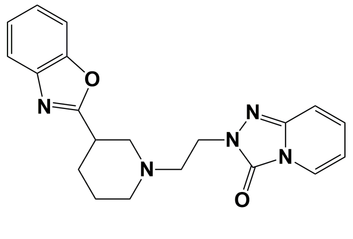
**98**	7156490114	- 7.8	+ 0.8 (± 0.4)	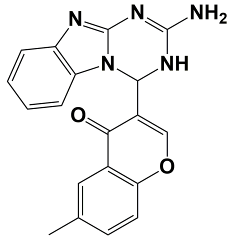
**99**	8401327893	- 9.1	- 1.0 (± 0.4)	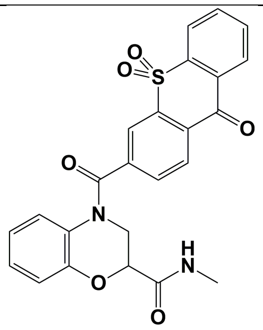
**100**	7691843385	- 9.0	+ 0.9 (± 0.2)	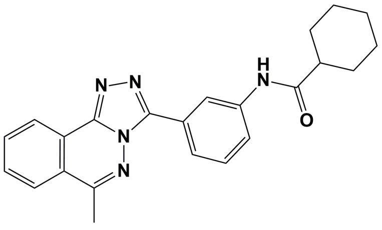
**101**	7494210657	- 8.8	+ 0.8 (± 0.3)	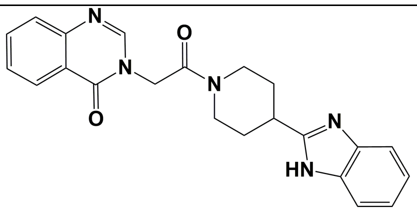

**102**	5933106976	- 7.9	+ 0.2 (± 0.2)	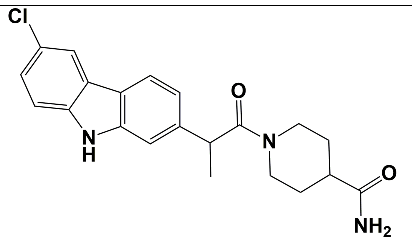
**103**	6743584260	- 7.6	+ 0.3 (± 0.3)	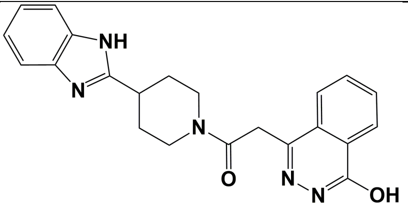

[a] $\Delta T_m = T_{m\ (TERRA\ G4+10\ \mathrm{ligand\ equiv})} - T_{m\ (TERRA\ G4)}$. The T_m value for *TERRA G4* in the absence of ligand was 74.3 (± 0.1) °C.

Figure S1. Circular dichroism spectra (01)

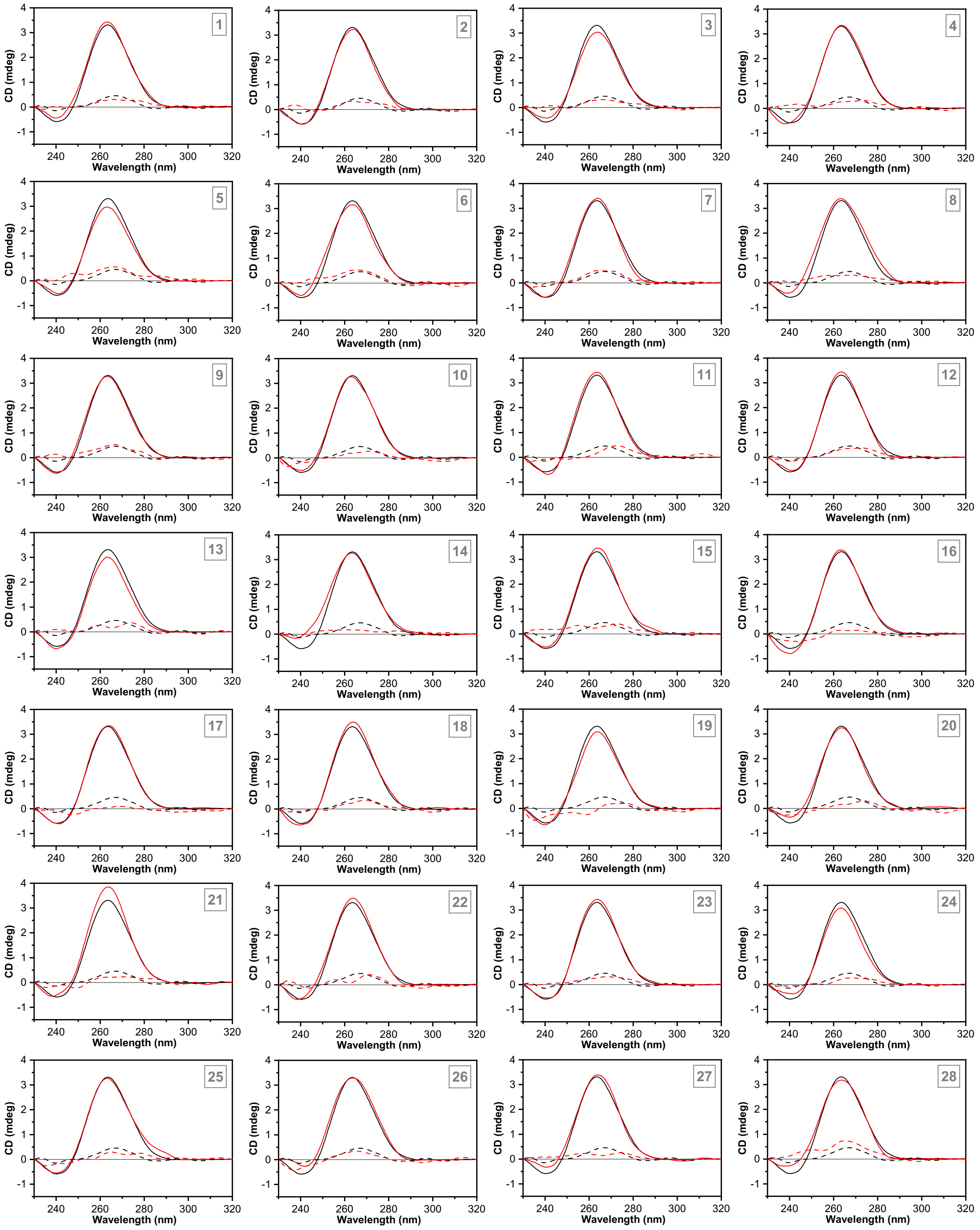

Figure S1. Circular dichroism spectra (02)

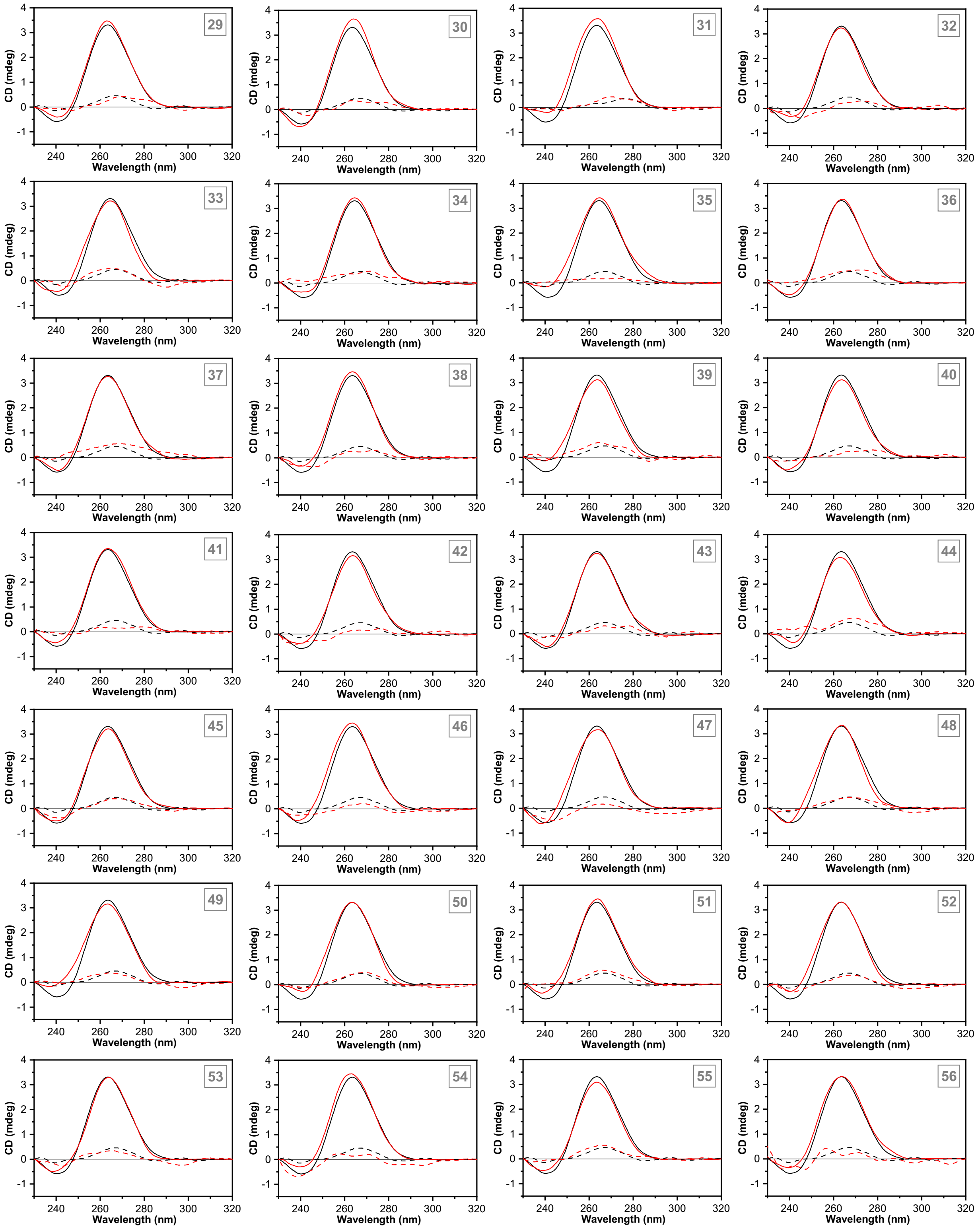

Figure S1. Circular dichroism spectra (03)

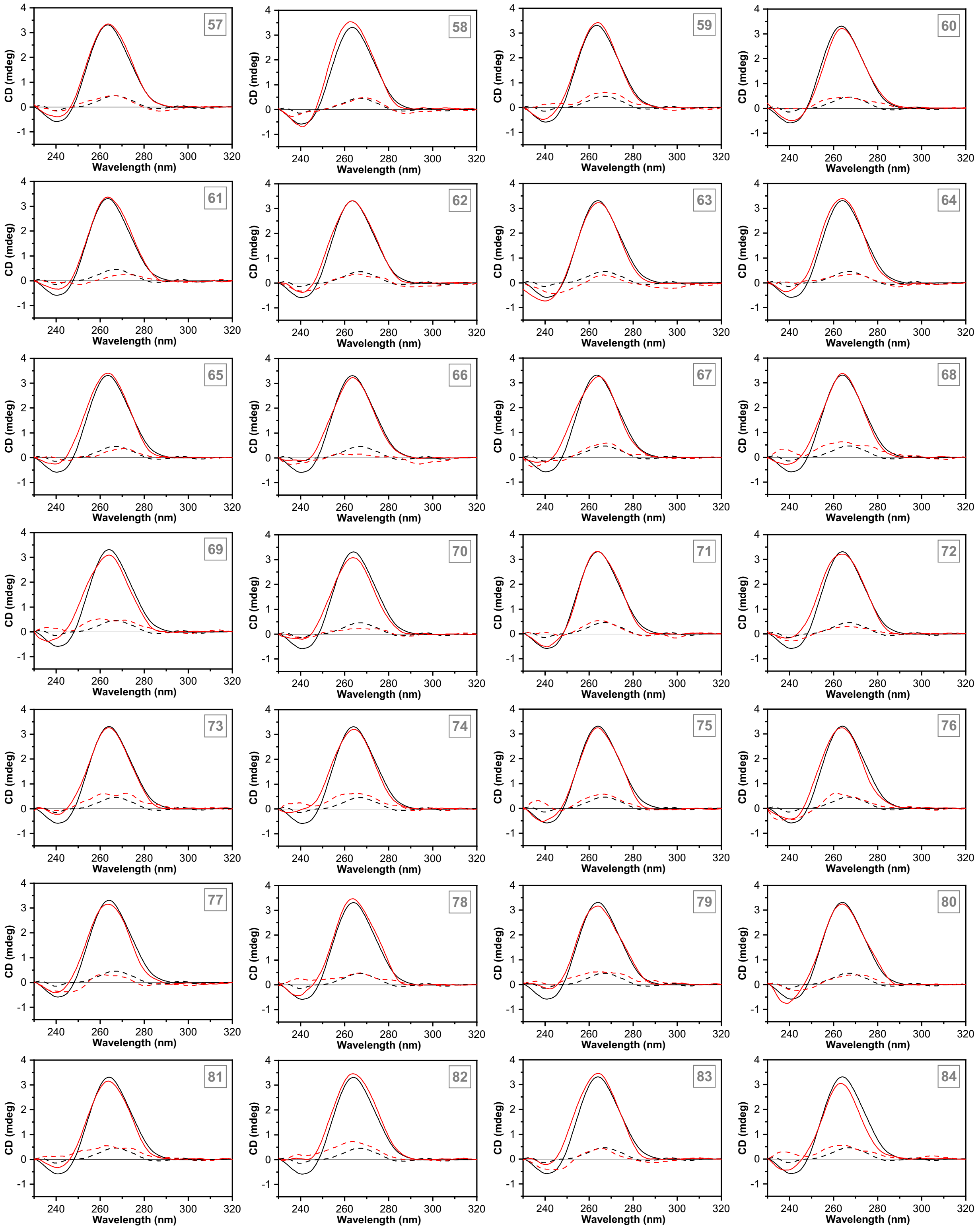

Figure S1. Circular dichroism spectra (04)

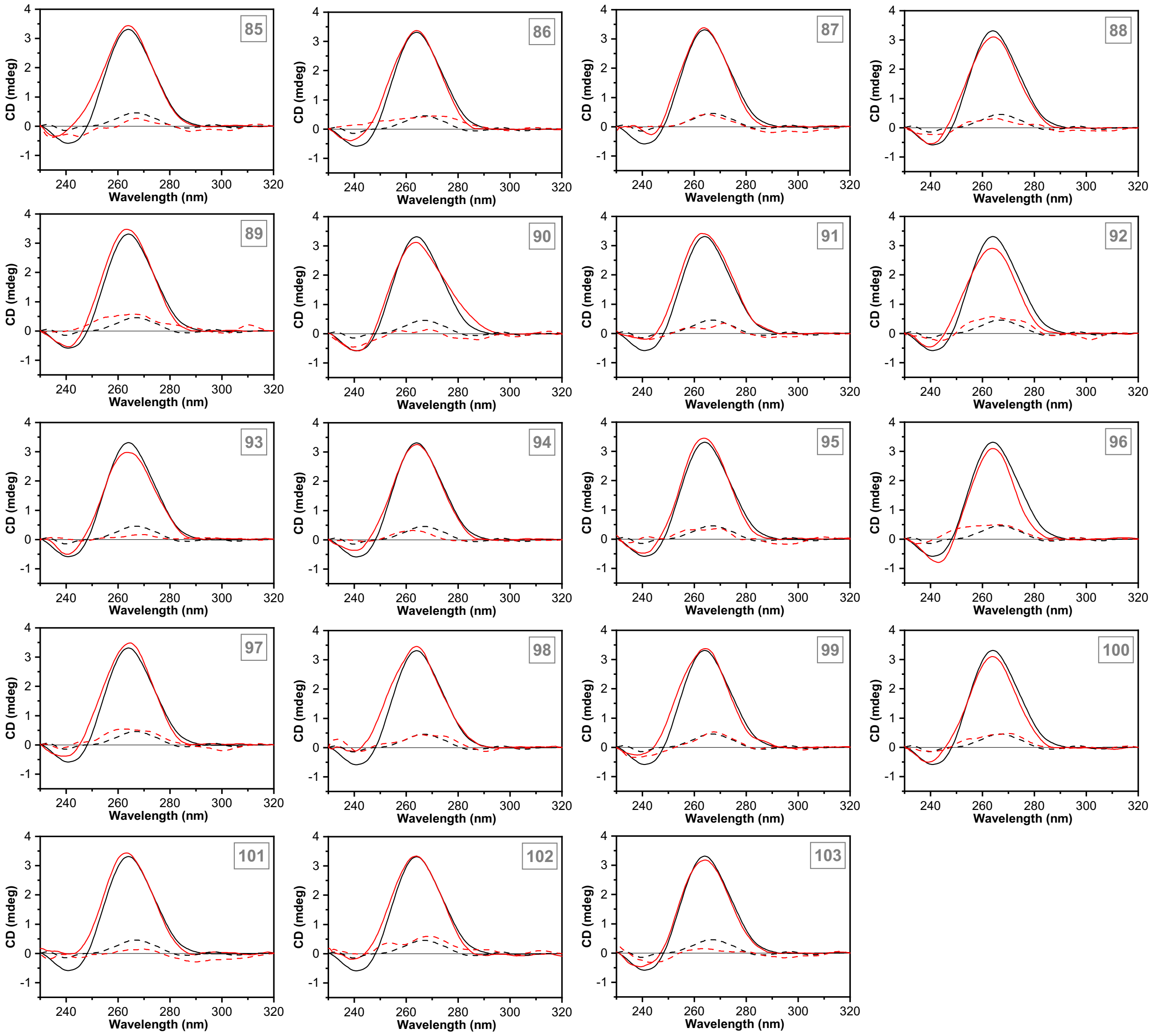

Figure S1. CD spectra of *TERRA G4* before (solid lines) and after (dashed lines) melting experiment (at 20 and 100 °C, respectively) in the absence (black) and in presence (red) of 10 molar equiv of compounds **1–103.**

Figure S2. Circular dichroism melting curves (01)

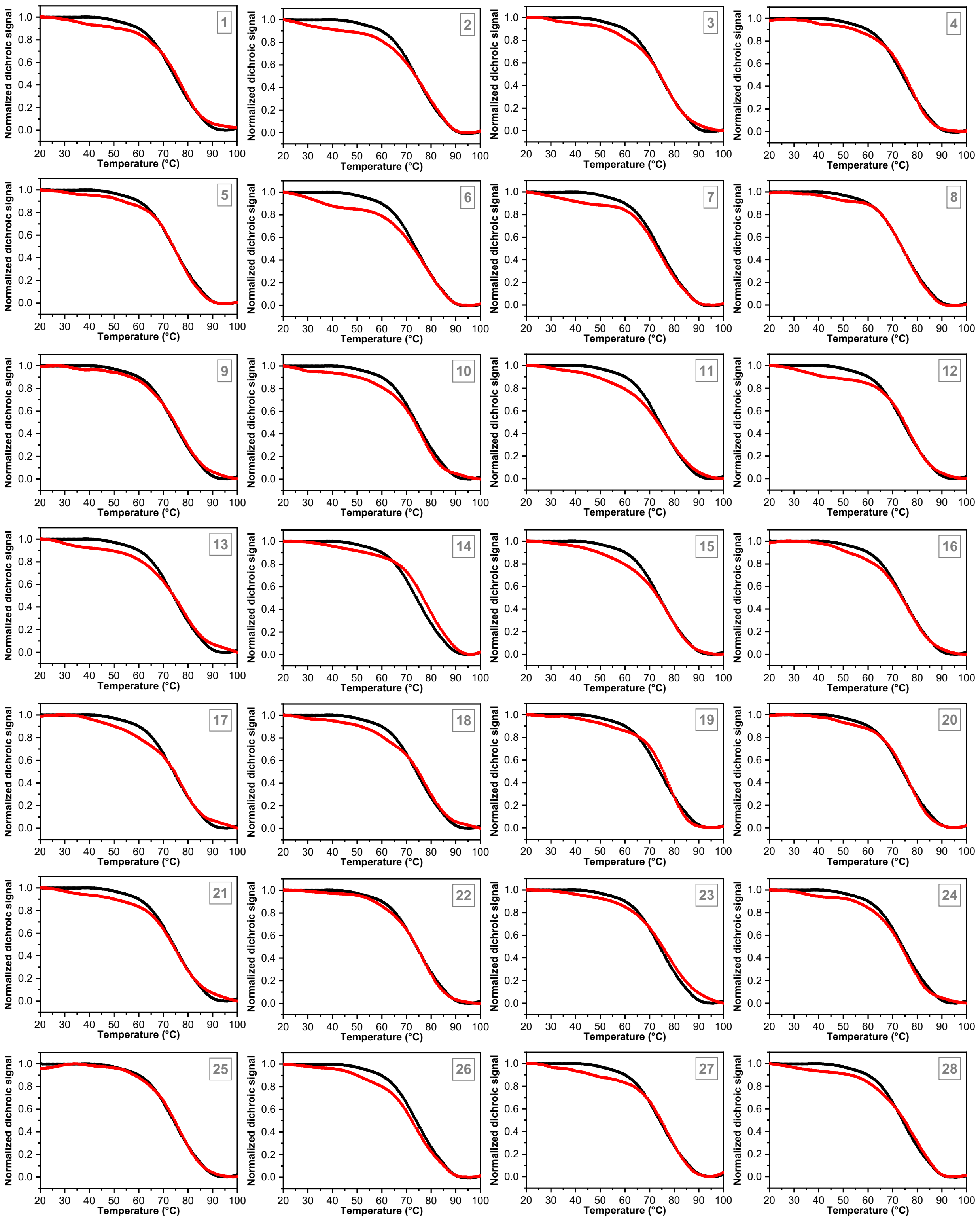

Figure S2. Circular dichroism melting curves (02)

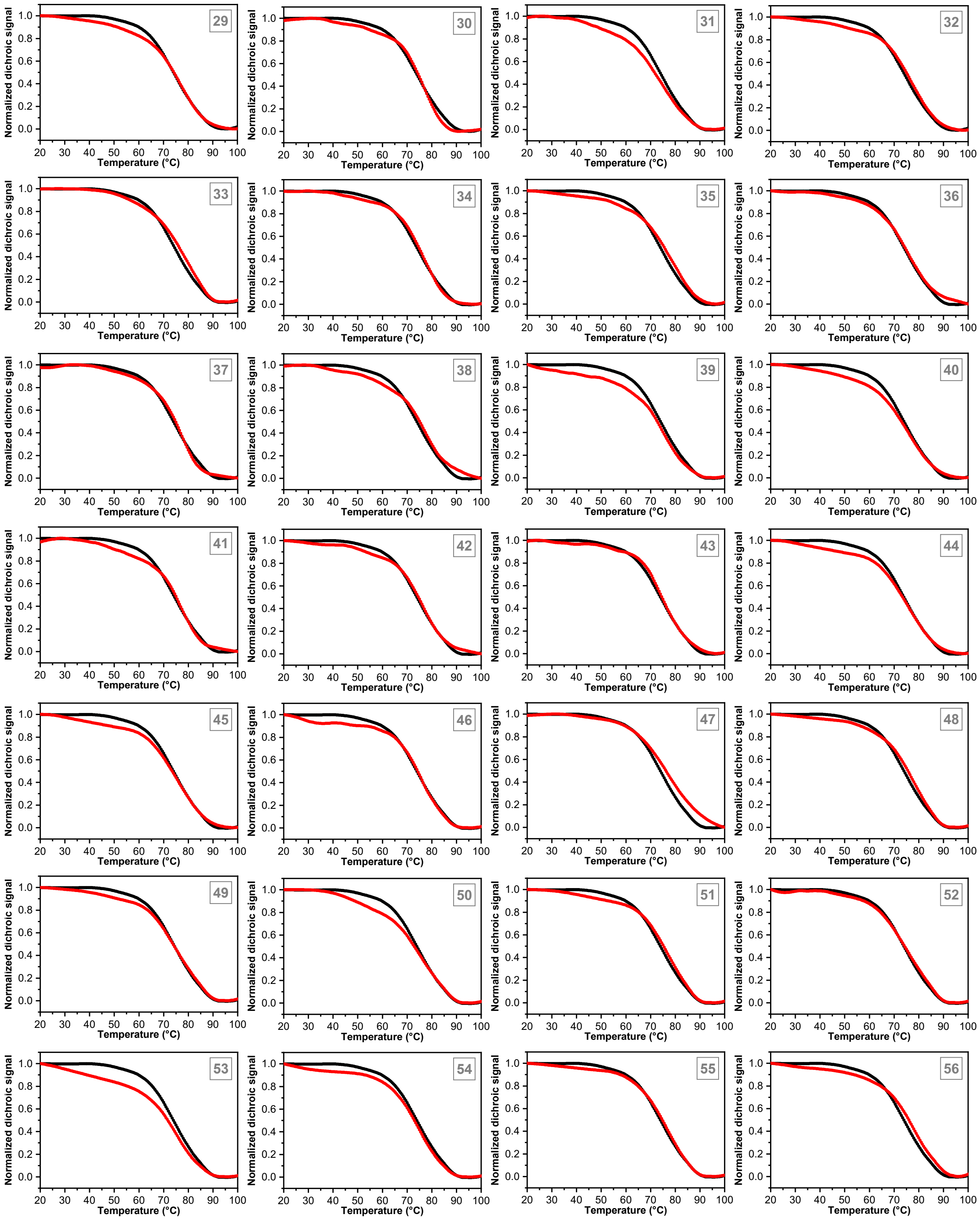

Figure S2. Circular dichroism melting curves (03)

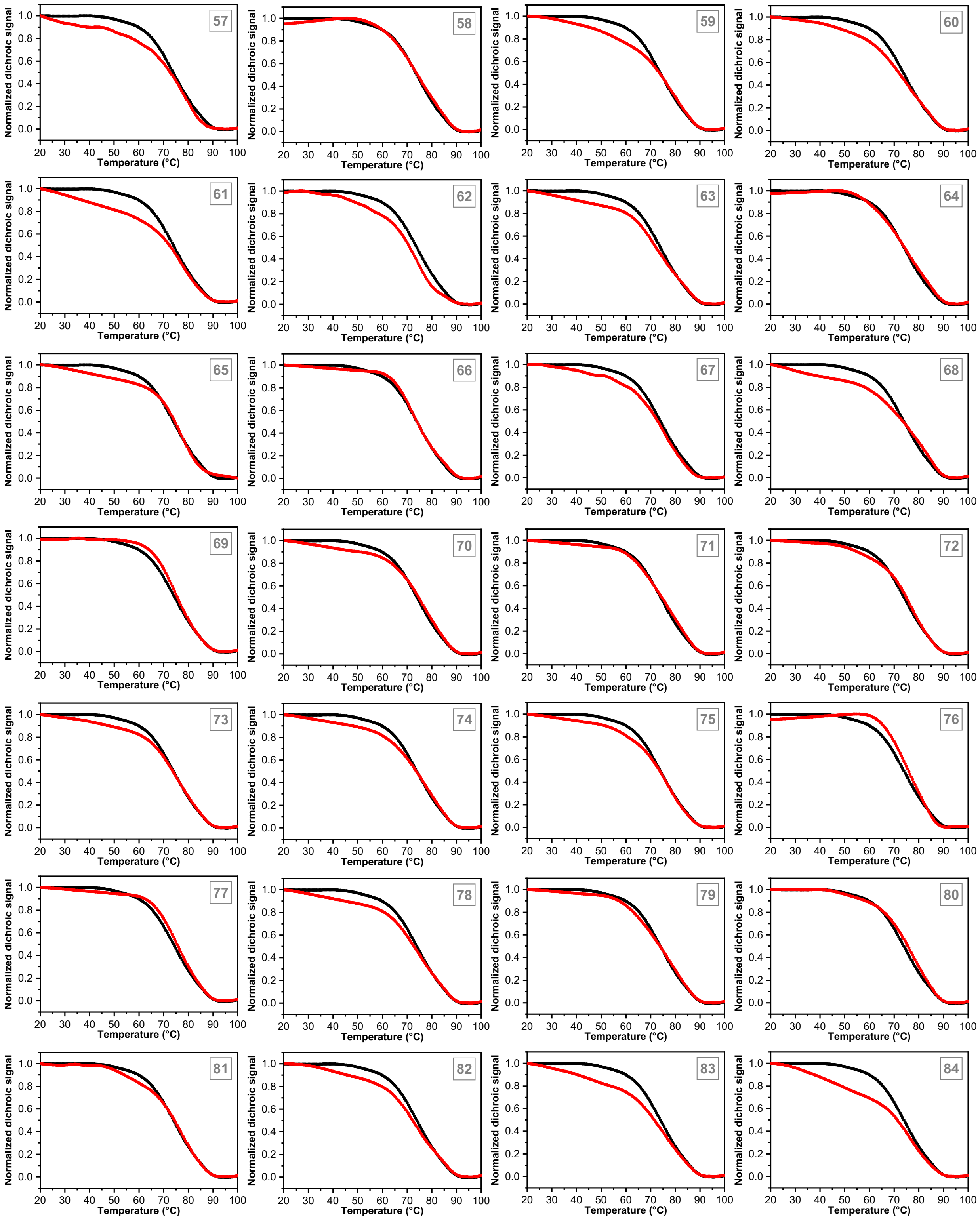

Figure S2. Circular dichroism melting curves (04)

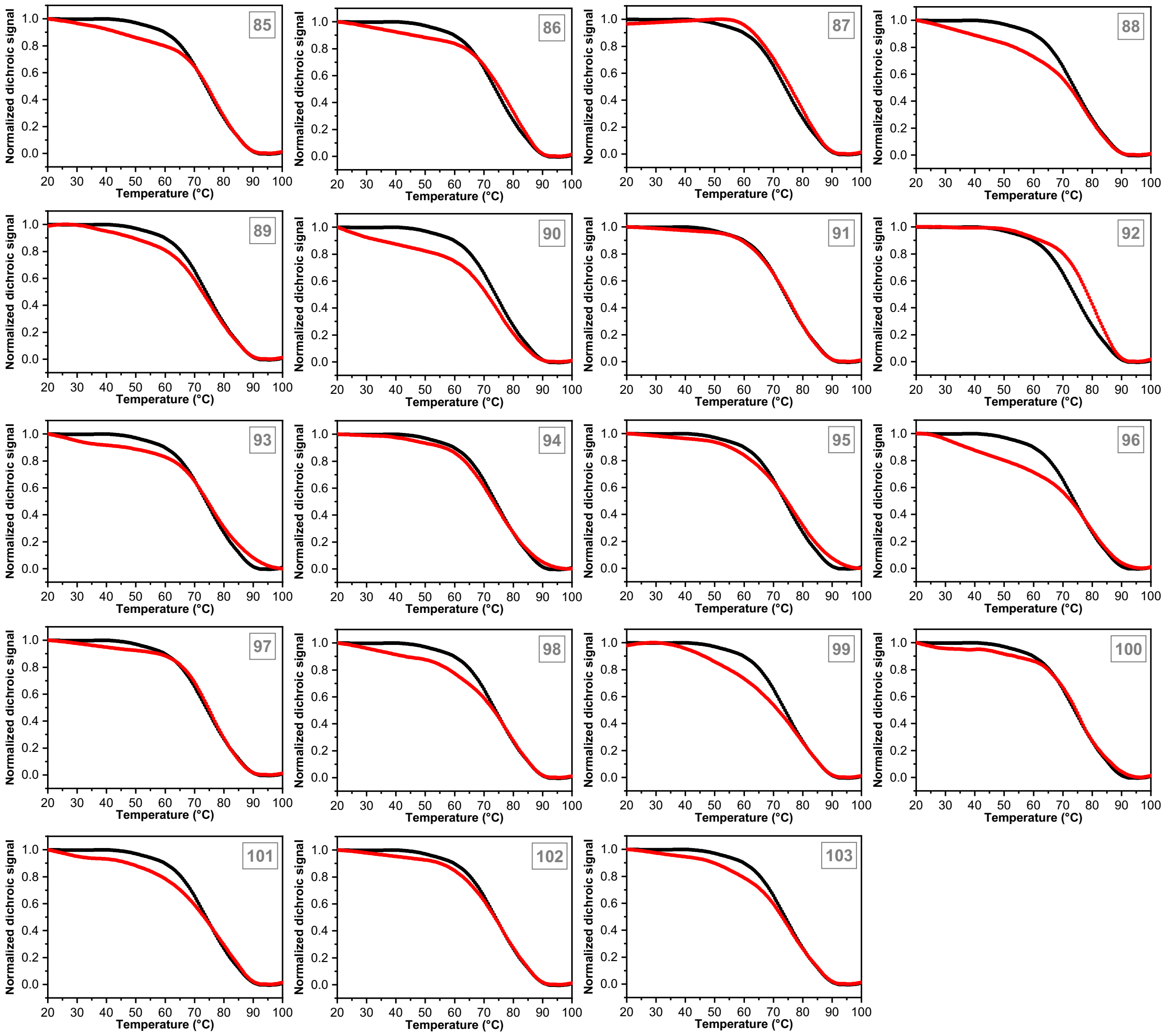

Figure S2. Normalized CD melting curves for *TERRA G4* in the absence (black) and in presence (red) of 10 molar equiv of compounds **1–103.**

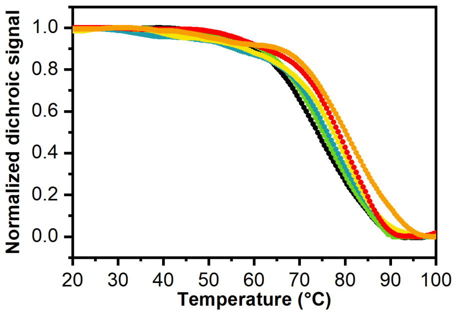

Figure S3. Normalized CD melting curves for *TERRA G4* in the absence (black) and in presence of 1 (blue), 2 (green), 5 (yellow), 10 (red), and 25 (orange) molar equiv of **BPBA**.

Table S2. Melting temperature variations for *TERRA G4* upon addition of increasing amount of **BPBA** determined by CD melting experiments.

[BPBA] μM	ΔT_m (°C)
2	+ 1.4 (±0.3)
4	+ 3.6 (±0.3)
10	+ 4.3 (±0.3)
20	+ 4.5 (±0.4)
50	+ 5.2 (±0.4)

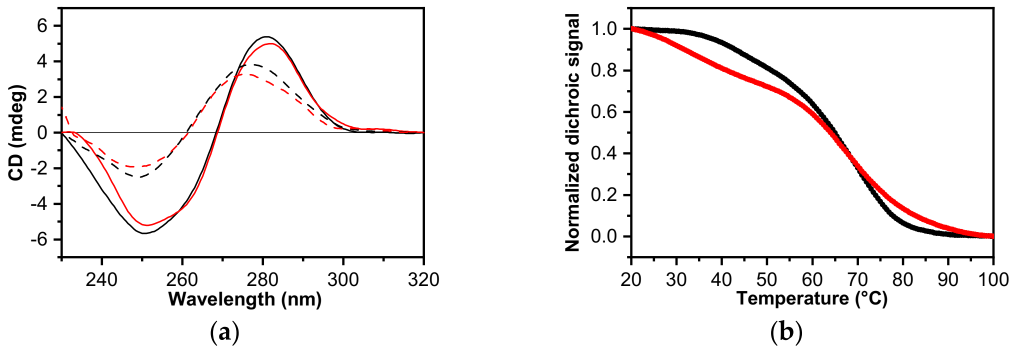

Figure S4. (**a**) CD spectra of *Hrp* in the absence (black line) and presence (red line) of 10 molar equiv of **BPBA** (entry **92**) recorded at 20 and 100 °C (solid and dashed lines, respectively). (**b**) normalized CD melting curves for *Hrp* in the absence (black) and presence (red) of 10 molar equiv of **BPBA** recorded at 1 °C/min heating rate.

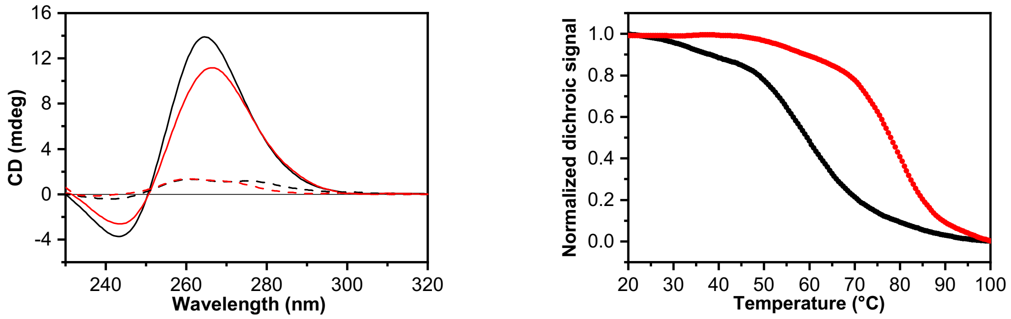

(**a**)

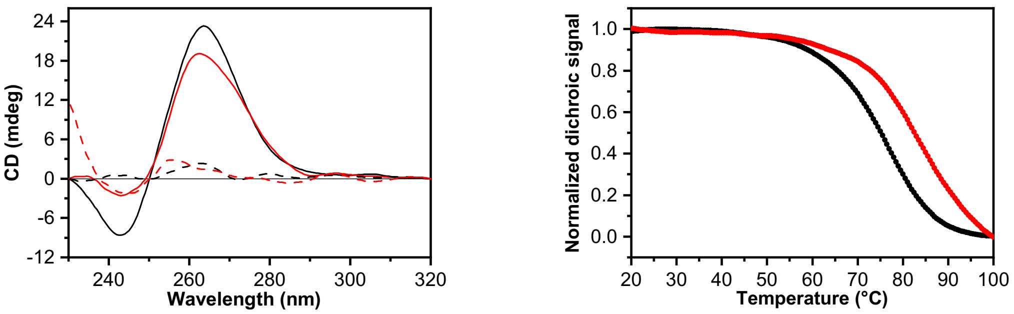

(**b**)

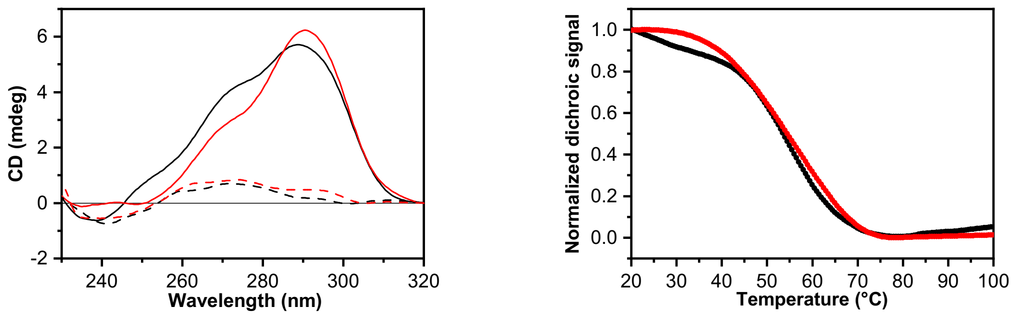

(**c**)

Figure S5. (Left) CD spectra of (**a**) *c-kit2 G4*, (**b**) *c-myc G4*, and (**c**) *Tel$_{23}$ G4* in the absence (black line) and presence (red line) of 10 molar equiv of **BPBA** (entry **92**) recorded at 20 and 100 °C (solid and dashed lines, respectively). (Right) Normalized CD melting curves for (**a**) *c-kit2 G4*, (**b**) *c-myc G4*, and (**c**) *Tel$_{23}$ G4* in the absence (black) and presence (red) of 10 molar equiv of **BPBA** recorded at 1 °C/min heating rate.

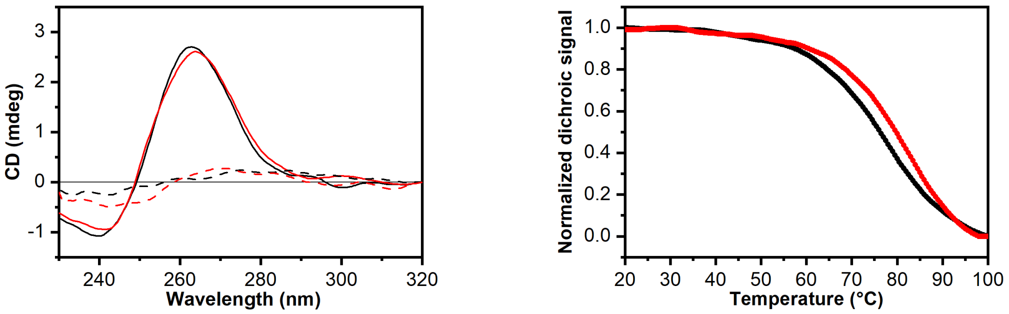

(**a**)

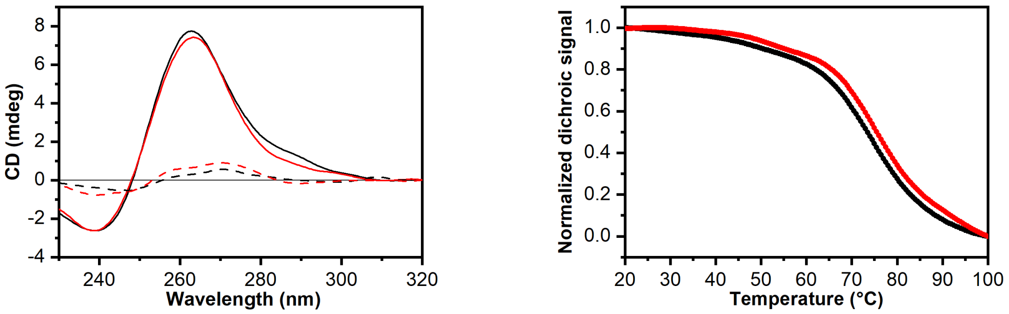

(**b**)

Figure S6. (Left) CD spectra of (**a**) *Bcl-2 G4* and (**b**) *GSEC G4* in the absence (black line) and presence (red line) of 10 molar equiv of **BPBA** (entry **92**) recorded at 20 and 100 °C (solid and dashed lines, respectively). (Right) Normalized CD melting curves for (**a**) *Bcl-2 G4* and (**b**) *GSEC G4* in the absence (black) and presence (red) of 10 molar equiv of **BPBA** recorded at 1 °C/min heating rate.

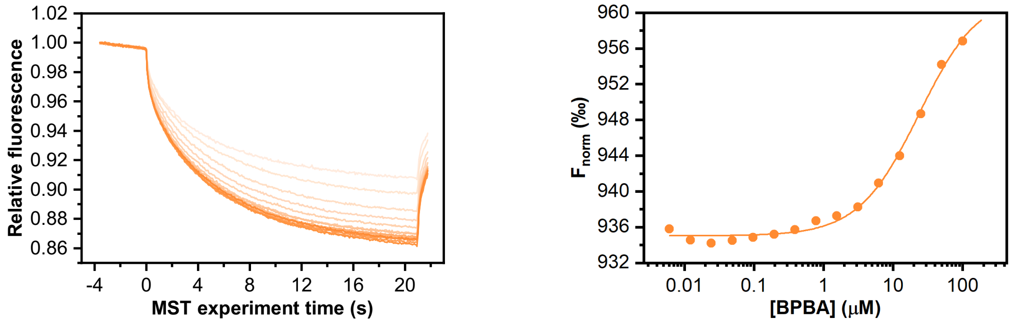

(**a**)

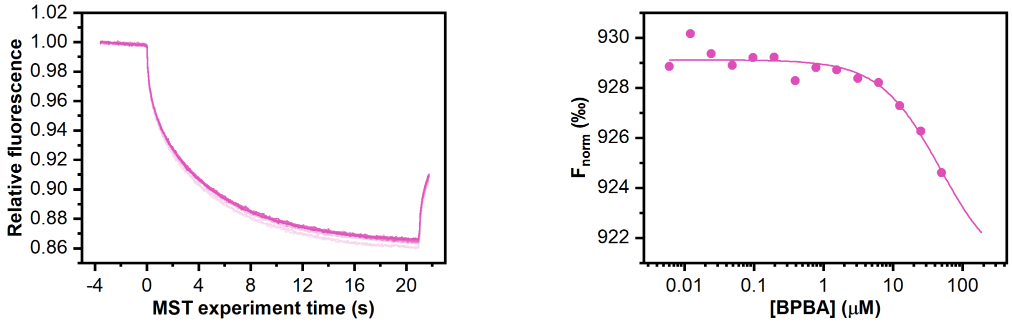

(**b**)

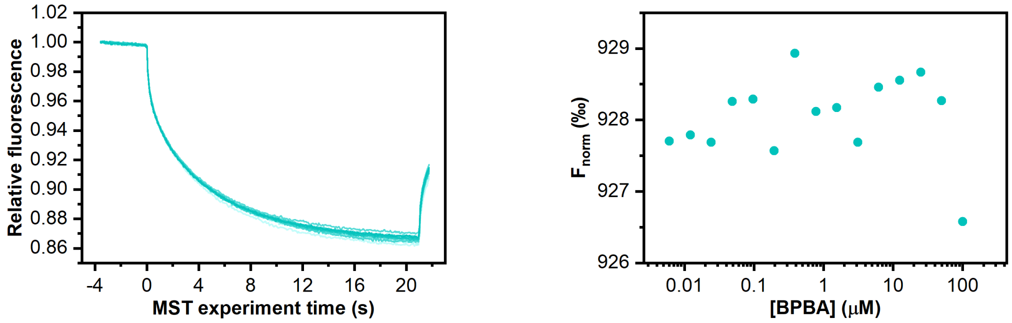

(**c**)

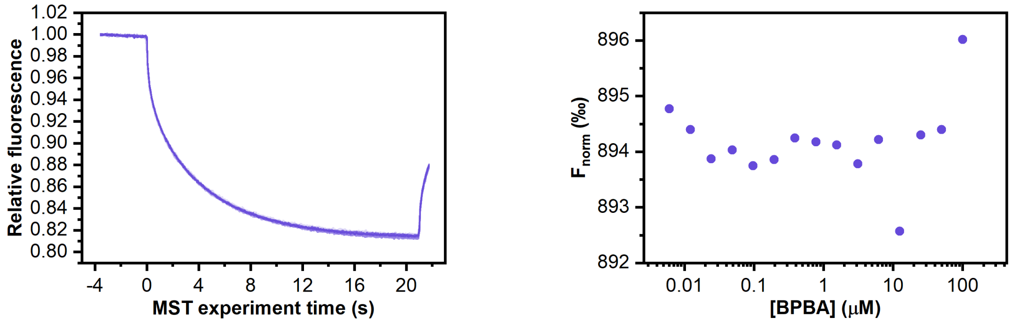

(**d**)

Figure S7. MST measurements for the interaction of **BPBA** (entry **92**) with (**a**) *c-kit2 G4*, (**b**) *c-myc G4*, (**c**) *Tel*$_{23}$ *G4*, and (**d**) *Hrp*. (Left) Time traces recorded by incubating increasing concentrations of compound with the labeled DNA molecules and (right) the corresponding binding curves.
